# Sequencing of the *Arabidopsis* NOR2 reveals its distinct organization and tissue-specific rRNA ribosomal variants

**DOI:** 10.1038/s41467-020-20728-6

**Published:** 2021-01-15

**Authors:** Jason Sims, Giovanni Sestini, Christiane Elgert, Arndt von Haeseler, Peter Schlögelhofer

**Affiliations:** 1 Department of Chromosome Biology, Max Perutz Labs, University of Vienna, Vienna BioCenter, Vienna, Austria; 2 Center for Integrative Bioinformatics Vienna (CIBIV), Max Perutz Labs, University of Vienna and Medical University of Vienna, Vienna BioCenter, Vienna, Austria; 3 Present Address: Institute of Molecular Biotechnology of the Austrian Academy of Sciences (IMBA), Vienna Biocenter (VBC), 1030 Vienna, Austria

**Keywords:** Plant genetics, Plant molecular biology

## Abstract

Despite vast differences between organisms, some characteristics of their genomes are conserved, such as the nucleolus organizing region (NOR). The NOR is constituted of multiple, highly repetitive rDNA genes, encoding the catalytic ribosomal core RNAs which are transcribed from 45S rDNA units. Their precise sequence information and organization remain uncharacterized. Here, using a combination of long- and short-read sequencing technologies we assemble contigs of the *Arabidopsis* NOR2 rDNA domain. We identify several expressed rRNA gene variants which are integrated into translating ribosomes in a tissue-specific manner. These findings support the concept of tissue specific ribosome subpopulations that differ in their rRNA composition and provide insights into the higher order organization of NOR2.

## Abstract (web summary)

The nucleolus organizing region (NOR) consists of multiple, highly repetitive rDNA genes. Here Sims et al. use both long- and short-read sequencing to determine the organization and sequence of Arabidopsis NOR2 rDNA and show that different rRNA gene variants are integrated into translating ribosomes in a tissue-specific manner.

## Introduction

Ribosomes are one of the oldest and most complex molecular machines in extant life. In all living cells, they are responsible for translating the genetic information residing on mRNAs into proteins. Ribosomes are large complex molecules, build from at least 80 proteins (estimate for *Arabidopsis*) and 4 types of ribosomal RNAs (rRNAs) that constitute the catalytic core^1^. In *Arabidopsis*, the 18S, 5.8S, and 25S rRNA genes are organized, in a head-to-tail manner, within large domains known as nucleolus organizing regions (NORs)^2,3^ located at the subtelomeric domains of chromosomes 2 and 4. A single rDNA unit contains three rRNA genes (18S, 5.8S, and 25S) with two internal and external transcribed sequences (ITS and ETS, respectively). In addition, each unit contains a core promoter sequence preceded by one or multiple spacer promoters flanked by SalI repeat boxes^4^. Several studies have shown that the rDNA repeats are not completely identical, with variability within the intergenic regions and, to some extent, also in the coding sequences^5–7^. It remains unknown whether these variations (VARs) are present in the translating ribosome and have a functional impact. Four main VARs are located within the 3′-ETS and grouped on separate chromosomes^6,8^. VAR1 and VAR3a, which are believed to be generally interspersed, are located on NOR2, whereas VAR2 and VAR3b-c are located on NOR4^8,9^.

Although the rRNA transcripts account for ~50% of all transcribed RNA in a cell, only a fraction of the tandemly repeated rDNA genes is transcribed at a given time^6,7^. The transcriptional regulation of individual rRNA genes and their organization within the NOR domains remains unknown, for any organism, due to their repetitive nature that hinders classical sequencing approaches^7,10^. In fact, it has never been possible to assemble the complete NOR by directly sequencing the genome of *Arabidopsis* or that of any organism. The current *Arabidopsis* genome assembly only contains single rDNA units at the top of chromosomes 2 and 4. Furthermore, none of the recent *Arabidopsis thaliana* genome assembly approaches^10,11^, performed with devices and protocols from Oxford Nanopore Technologies or PacBio, provided contigs with multiple 45S rDNA units in tandem. The difficulty in assembling the NORs, from whole-genome (WG) sequencing projects, is intrinsic to the low complexity of the nearly identical rDNA units. Currently, ultra-long reads are not available in sufficient length and number to unambiguously reconstruct the rDNA regions on chromosome 2 and 4 directly from plant DNA. An in-depth understanding of the regulation and organization of the rDNA repeats would require the analysis of the full sequence of the NORs.

In this study, we used a combination of long- and short-read sequencing to obtain the exact sequence information of 405 individual rDNA repeats and their arrangement. We sequenced 59 bacterial artificial chromosomes (BACs) containing ~7 rDNA repeats each and devised a sophisticated barcoding system to align each BAC and build long rDNA contigs. This allowed us to assess the higher-order organization of *A. thaliana*’s NOR2 and build its first sequence draft. We identified several sequence VARs in the rRNA genes and established that the corresponding rRNAs are expressed and integrated into mature and translating ribosomes in a tissue-specific manner. These findings uncover a complex transcriptional regulation of rDNA genes and support the concept of tissue-specific ribosome subpopulations^12^ that differ in their rRNA composition.

## Results

### Sequencing of the NOR2 rDNA

**Fig. 1:**
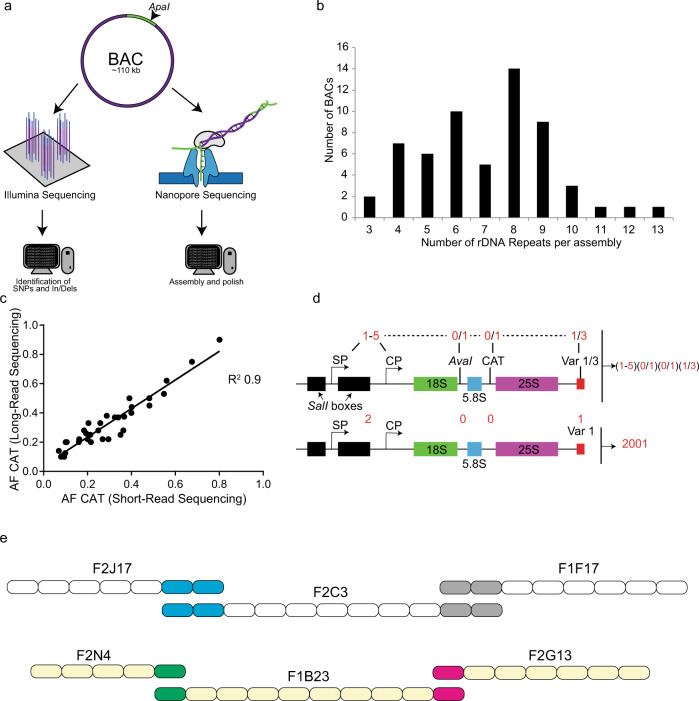
Sequencing and polishing BACs containing rDNA contigs. **a** Scheme depicting the sequencing and assembly strategy combining long-read (NanoPore) and short-read sequencing (Illumina) of BACs containing rDNA genes. **b** Graph depicting the number of rDNA units per assembled BAC. **c** Correlation analysis of the CAT allele frequencies derived from the long- and short-read sequencing approaches. Each dot represents an individual assembly. **d** Graphical description of the numeric codes attributed to the described rDNA unit features. Each four-digit code corresponds to a unique rDNA variant. An example is shown of an rDNA variant that carries 2 promoters (1 spacer promoter, SP and 1 core promoter, CP), no AvaI site (0), no CAT insertion (0), and the 3′-ETS VAR1. It is defined as a “2001” rDNA repeat. **e** Schematic representation of the overlap between BACs F2J17-F2C3-F1F7 and F2N4-F1B23-F2G13. Each rDNA units is defined by a rectangle with the four feature barcode and the SalI box barcode in parenthesis.

Our aim was to generate a draft assembly of the NOR2 of *A. thaliana*. To successfully sequence and build NOR2, we relied on a BAC-based approach^13^ (Fig. 1a). BACs, initially generated to obtain the *Arabidopsis* physical map^14^, containing rDNA repeats were ordered from the Arabidopsis Biological Resource Center (ABRC) stock center^15^. They were classified according to the type of 3′-ETS variant (VAR), to discriminate their origin from either NOR2 or NOR4^8^ (Supplementary Fig. 1a). BACs containing rRNA genes with VAR1 or VAR3 were further processed and sequenced to construct a draft of the NOR2 domain. Supercoiled BACs from bacterial cultures were linearized with the restriction enzyme ApaI prior to sequencing and analyzed by pulse-field gel electrophoresis (PFGE) (Supplementary Fig. 1b).

In total, we sequenced 59 BACs with an average BAC assembly size of about 77 kb (Supplementary Table 1) with Oxford Nanopore Technologies. We used reads longer than 50 kb for the assembly of the BACs (Supplementary Fig. 1c). The assemblies contain an average of seven rDNA repeats of ~10 kb each. The assembly sizes of 49 out of 59 BACs are coherent with the expected sizes according to the PFGE analysis^15^ (Supplementary Fig. 1b) and contain the vector backbone separated at the ApaI cut site. These BACs were considered as fully assembled. The number of rDNA repeats per assembly is depicted in Fig. 1b. In parallel, the 49 fully assembled BACs were also sequenced by short-read sequencing technology (Illumina), to confirm single-nucleotide polymorphisms (SNPs) and insertions and deletions (InDels) (Supplementary Table 1). In the assemblies analyzed, the experimentally determined length of the SalI boxes matched the expected sizes (Supplementary Fig. 1d). BAC assembly F2C3 after restriction digest released fragments of ~4700 and ~3900 bp, F1E12 and F2G13 released fragments of ~3500 and ~1500 bp, and F2G18 released fragments of ~1500 bp (Supplementary Fig. 1d). Furthermore, the cleaved amplified polymorphic sequence (CAPS) analysis confirmed the presence of the polymorphism at the sites anticipated from the assemblies (Supplementary Fig. 1e). Finally, for the full assemblies (49 BACs), the allele frequencies (AFs) determined by short-read sequencing matched in number and nature those determined in the assembled BACs (Supplementary Data 1). For instance, the *R*^2^ correlation between the SNP/InDel frequencies of the long-read assemblies with the short-read sequences for the AvaI restriction site and the trinucleotide insertion “CAT” polymorphisms^7^ (position 4466 of the reference rDNA) is 0.74 and 0.9, respectively (Fig. 1c, Supplementary Fig. 1f, and Supplementary Table 1). In all assemblies, the rDNA repeats are organized in a head-to-tail manner as shown previously^3^. This latter finding substantiates the quality and reliability of our sequencing efforts, arguing against a possible re-arrangement of the multiple rDNA repeat units present in the BACs during library generation or faulty assembly. To confirm that the SNPs and InDels identified in the 49 fully assembled BACs were present in the corresponding genome of *A. thaliana* Col-0 plants, we made use of WG sequencing data previously generated in our lab (Kurzbauer et al. (2020), submitted). This data set was obtained by Illumina 125 bp paired-end sequencing and yielded a genome-wide 30-fold coverage. The reads corresponding to the rDNA were mapped to the reference rDNA repeat (Supplementary Data 2) and SNPs/InDels called with LoFreq. We obtained the nature and frequency of occurrence of SNPs and InDels of all rDNA units present in the genome, located on both NOR2 and NOR4. We only took into consideration AFs above 0.125% (frequencies obtained if one SNP/InDel would be present in a single rDNA unit out of ~800 units), as it is estimated that the *Arabidopsis* genome contains ~800 rDNA repeats^2^. Fifty-eight percent of the SNPs/InDels in the BACs were also present in the genome (Supplementary Data 2 and 3). The remaining 42% of SNPs/InDels that were not found in the genome could represent genetic background alterations, which may have arisen from mutagenic events during the generation of the BAC library or are false positives called by LoFreq (Supplementary Data 3). We were able to successfully assemble and confirm 49 BACs, which contained an average of 7 rDNA units per BAC. These assemblies provided the base to build the first draft of the *Arabidopsis* NOR2.

### rRNA gene variants guide the assembly of NOR2

To guide the building of a first draft of the NOR2 domain, we devised two “barcode” systems to discriminate each rDNA repeat unit, which are 99% identical. The first barcode system is based on four markers as follows: (1) number of core and spacer promoters^5^, (2) presence of the AvaI restriction site (at position 4133 of the reference rDNA)^9^, (3) presence of a specific trinucleotide (CAT) insertion (at coordinate 4466 of the reference rDNA)^9^, and (4) length of the 3′-ETS^6^. This barcode system allows us to individualize each rDNA unit and guide the overlap between BAC assemblies generating larger contigs. The second barcode system is based on the length and number of the SalI boxes^5^. It has been used separately due to the highly complex nature of the SalI boxes.

The number of promoters, the length of the 3′-ETS, and the SalI boxes were selected as markers, as they largely vary between different rDNA repeats^5,6,16^. The AvaI restriction site is equally distributed between NOR2 and NOR4, whereas the CAT marker was shown to be strongly enriched on NOR2^8^. All other SNPs/InDels were not taken into consideration, as they are either of low allelic frequency or are associated to the markers mentioned above. The “four marker barcode system” shows that the most frequently occurring unit type is 2001 (38%) (2 promoters, the absence of AvaI, the absence of CAT, and 3′-ETS variant 1) (Table 1), 11% are of type 2003, 10% are of type 3001, 8.6% are of type 3111 (Fig. 1d and Supplementary Fig. 2a). For the “SalI box barcode system,” each rDNA repeat was categorized by an alphanumeric code with each letter corresponding to a specific SalI box length. rDNA units with multiple letters indicate the presence of multiple SalI boxes of a given type (Table 1). The most frequent SalI box combinations are EZ (14%), EU (12.5%), EV (11%), EEV (8.5%), EEU (8.3%), FQ (6%), I (5.8%), and M (5.8%) (Supplementary Fig. 2b).

**Table 1:** Glossary for the alphanumeric feature classification.

CODE	Features
1–5	Promoters
0/1	AvaI
0/1	CAT
1/3	VAR
A	310 bp Deletion
B	330 bp Deletion
C	270 bp Deletion
D	334 bp Deletion
**CODE**	**SalI boxes**
D	SalI 200–249 bp
E	SalI 250–299 bp
F	SalI 300–349 bp
I	SalI 450–499 bp
M	SalI 550–599 bp
&	SalI 600–649 bp
O	SalI 700–749 bp
Q	SalI 800–849 bp
R	SalI 850–899 bp
T	SalI 950–999 bp
U	SalI 1000–1049 bp
V	SalI 1050–1099 bp
W	SalI 1100–1149 bp
Z	SalI 1250–1299 bp
Ψ	SalI 1450–1499 bp
λ	SalI 1500–1549 bp
ρ	SalI 1800–1850 bp

Glossary of the different features used to individualize each rDNA unit.

We analyzed the individual rDNA units and found that units containing the CAT insertion and the EEV or EEU SalI box type are associated in 60% of all cases with the AvaI site. In contrast, rDNA units containing the CAT and the M or FQ SalI box types are devoid of the AvaI site in all cases. Interestingly, the AvaI site is in 99% of all cases associated with the CAT insertion site (Supplementary Fig. 2c). A small fraction (≤5%) of the rDNA units contained large deletions spanning 270–334 bp located within both the transcribed and non-transcribed regions (Supplementary Fig. 3a–c). These deletions are located within the 5′-ETS (A and B), the 18S rRNA^17^ (C), and spanning the 18S and the ITS1 (D) (Supplementary Figs. 2a and 3a–c). This initial sequence analysis and the establishment of the barcode systems gave each rDNA unit an identity and provided a solid base to overlap BAC assemblies with high confidence.

To determine possible overlapping BAC assemblies, we first designed a program (OverBACer) that determines, for each assembly, the sizes of the SalI boxes, the types of the 3′-ETS variants present, and the distances (in bps) between them. The program generates a string of marker identities and distances creating a simple and unique identifier for each assembly. Subsequently, the assembly-specific strings were used to find matches between the different assemblies. The matching overlaps were manually confirmed by evaluating the AvaI and CAT markers. OverBACer automatically generated 53 unique contigs using the sequencing input from 59 BACs, with an average length of ~80 kb. Ten assemblies were found to overlap with high confidence, with 230 kb as the longest contig (derived from BACs F2J17-F2C3-F1F17) (Fig. 1e). The other contigs of 200, 90, and 80 kb derived from BACs F2N4-F1B23-F2G13, F2G18-F19A6, and F1F11-F1N7, respectively (Fig. 1e). The relative low amount of overlaps identified between the 59 BACs led to the assumption that the NOR2 is much larger than previously anticipated (~400 repeats)^3^. The quality and length of the contigs generated provided a first draft of NOR2 that could be analyzed for its higher-order organization albeit of being incomplete.

### NOR2 is organized in distinct rDNA unit clusters

**Fig. 2:**
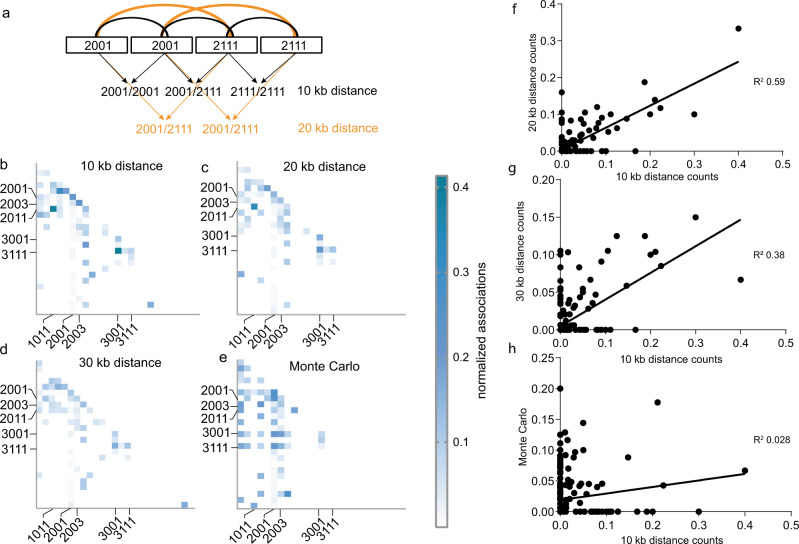
NOR2 is organized in distinct rDNA clusters. **a** Graphical description of the NeighbourFinder program. It counts the occurrence of rDNA unit types at a 10 kb (immediate neighbor), 20 kb (one unit downstream), or 30 kb (neighbor two units downstream) distance. **b**–**e** Heat maps depicting the number of neighboring rDNA unit types at a 10, 20, or 30 kb distance normalized to the sum of the rDNA units bearing the given features. Dark blue colors depict the highest relative number (0.42) and white the lowest (0). rDNA variants are listed as follows for each heat map: on the *y*-axis from top to bottom and for the *x*-axis from the left to right: 1001, 1003, 1011, 1013, 1111, 2001, 2003, 2011, 2013, 2101, 2111, 2113, 3001, 3011, 3111, 4001, 4111, 5001, 1003D, 1011D, 1013D, 2001A, 2001C, 2001B, and 2003C, (A, B, C, and D depict large deletions, see Table 1 for glossary) Most abundant ones are indicated. **f**–**h** Scatter plots showing the correlation between the frequencies of associated rDNA units of the 10 kb distance with the 20 kb distance (*R*^2^ = 0.59) (**f**), 10 kb distance with 30 kb distance (*R*^2^ = 0.38) (**g**), and the 10 kb distance with the numbers obtained by the Monte Carlo simulation (*R*^2^ = 0.028) (**h**).

Our sequencing results revealed that NOR2 is composed of heterogeneous rDNA units. We therefore evaluated whether the individual rDNA units, distinguished by their sequence features, are organized non-randomly. We designed a program (Neighbor Finder) that identifies rDNA units according to their features and counts the occurrence of neighboring rDNA unit types. The “four marker barcode system” described above was used for the analysis (Fig. 1d and Table 1). We classified rDNA units according to the selected features and analyzed co-occurring neighbors at 10, 20, or 30 kb distances, corresponding to the immediate neighboring unit, one further downstream and two further downstream, respectively (Fig. 2a, Supplementary Data 1). A Monte Carlo simulation (see “Methods”) of all rDNA units, maintaining the head-to-tail arrangement and the BAC conFig.uration, was generated as a control. Indeed, co-occurrences of some barcode pairs in the contigs are more frequent than expected from a random distribution. The analysis revealed that rDNA unit pairs (2011, 1011), (3111, 3001), and (2001, 2001) frequently co-occur in a distance of 10 and 20 kb. In contrast, rDNA unit pairs (2001, 3001) and (2001, 3111) never co-occur in a distance of 10, 20, and 30 kb, while they do so in the random simulation (Fig. 2b–e and Supplementary Data 1).

These results underline that rDNA unit types are not randomly distributed along NOR2 and that they form clusters. To illustrate this, we plotted the frequency of co-occurring pairs identified at 10 kb distance against the frequency of corresponding pairs counted at 20 kb, 30 kb distance, or in the random simulation. The *R*^2^ values clearly indicate correlations at 20 kb (*R*^2^ 0.59) and 30 kb (*R*^2^ 0.38), but not when compared to the frequency of pairs obtained from the Monte Carlo simulation (*R*^2^ 0.028) (Fig. 2f–h). Our results are in line with the findings obtained by Copenhaver et al.^3^ that suggested the existence of rDNA sub-clusters.

We also performed such an analysis after classifying rDNA units according to their SalI boxes. We find rDNA units harboring one, two or three SalI boxes of a similar type in close proximity to one another at a 10, 20 (*R*^2^ 0.52), and 30 (*R*^2^ 0.38) kb distance (Supplementary Fig. 4a). These results are different from the simulated data set where the units form distinct clusters not present in the experimental data (Supplementary Fig. 4b–d). Accordingly, the frequencies of co-occurring SalI boxes from the simulated data do not correlate with those obtained within a 10 kb distance (*R*^2^ 0.005) (Supplementary Fig. 4d).

The rDNA units found to co-occur following the analysis with four markers correlate well with those identified by the analysis using the SalI boxes. This represents a good internal control of our analysis as the number of promoters is proportional to the number of SalI boxes, as also shown previously by Havlova et al.^5^. These results suggest a higher-order organization of NOR2, highlighting that rDNA units with similar features tend to form clusters.

**Fig. 3:**
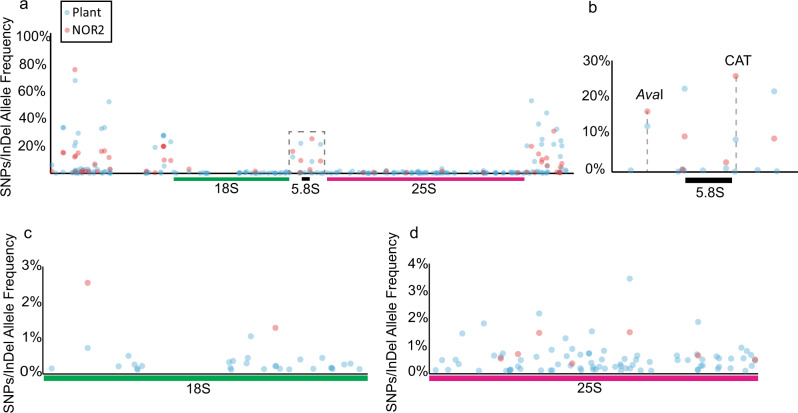
rDNA variants are differentially distributed between NOR2 and NOR4. **a** Plot depicting the SNP/InDel allele frequencies in rDNA units derived from genomic plant DNA sequencing (blue dots; representing allele frequencies in both NOR2 and NOR4) and from BAC sequencing (red dots; representing allele frequencies only in NOR2). Dashed box indicates the enlarged area depicted in **b**. **b** 5.8S rDNA region with the AvaI and the CAT alleles highlighted. **c** Allele frequencies in the 18S rDNA region. **d** Allele frequencies in the 25 S rDNA region.

The higher-order organization and the analyses of the 405 rDNA units of NOR2 provide a solid base for interpreting genomic Illumina short-read sequences deriving from WG sequencing experiments. The AFs of rDNA SNPs and InDels derived from short-read Illumina sequencing of the BACs can be related to the AFs derived from short-read sequencing of the WG of *Arabidopsis*. This allows attributing SNPs and InDels specifically to one NOR and determine the allelic proportion for those that occur in both. Polymorphisms found on both NOR2 and NOR4, at similar frequency, would have the same allelic frequency in the BAC-based Illumina sequencing (NOR2) and in the WG sequencing (plant), whereas polymorphisms more frequent on NOR2 than NOR4 have a higher allelic frequency in the BAC-based Illumina sequencing than in the WG sequencing and vice versa. The largest fraction of SNPs/InDels are present within the non-coding regions of the rDNA units as previously shown^7^ with most VARs present on NOR4 (plant) (Fig. 3a). One VAR that is prominently enriched on NOR2 is the CAT insertion at position 4466 with ~9% AF in plant-derived sequences and ~25% in the BAC-derived sequences (representing only NOR2) (Fig. 3b and Supplementary Data 2). Our results confirm the previous observation made by Chandrasekhara et al.^9^. We could detect that SNPs within the 25S rDNA are mainly located on NOR4 (Fig. 3d and Supplementary Data 2) in contrast to SNPs/InDels within the 18S, which are located on NOR2 (Fig. 3c and Supplementary Data 2). The differential distribution of SNP/InDels between the two NORs and the higher-order organization (clustering) of the rDNA units led us to design a specific fluorescence in situ hybridization (FISH) probe that would predominantly mark NOR2.

### Visualizing the CAT cluster on NOR2

**Fig. 4:**
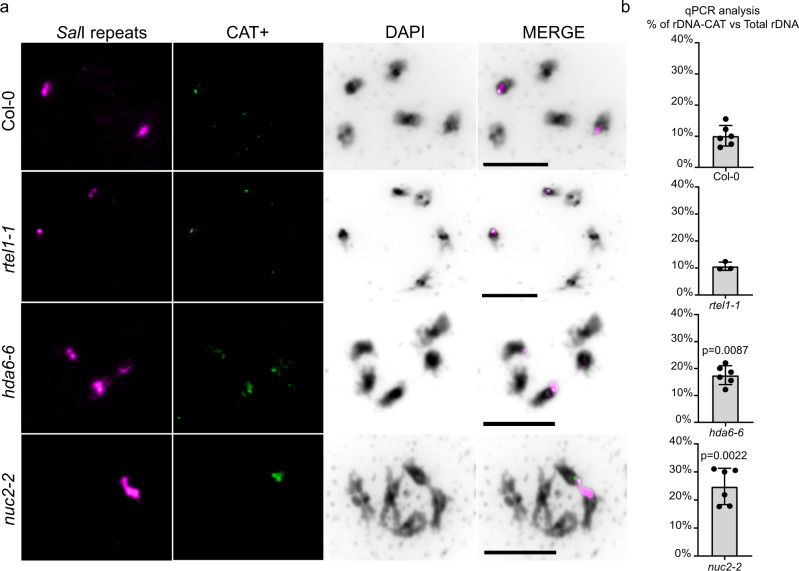
Visualizing the rDNA-CAT cluster on NOR2. **a** Spread nuclei from pollen mother cells at diakinesis or metaphase I stages from wild type (Col-0) and mutant lines (*rtel1-1*, *hda6-6*, *nuc2-2*) hybridized for the SalI repeats (magenta), the CAT insertion (CAT+, green), and stained with DAPI (black). The experiment was repeated three times with similar results. **b** Graphs of qPCR experiments from wild type (Col-0) and indicated mutant lines depicting the percentage of rDNA repeats bearing the CAT insertion related to the total amount of rDNA repeats. There is a statistically significant (*p* ≤ 0.001) increase in rDNA-CAT copy number in *hda6-6* and *nuc2-2* mutant lines. Statistical analysis was performed using the Mann–Whitney test. Error bars represent the SD.

To prove the existence of the rDNA unit sub-clusters, we designed a FISH experiment that targets and marks a specific rDNA sub-cluster on condensed (meiotic) chromosomes. One of the most prominent sequence features of the rDNA units located on NOR2 is a characteristic “CAT” insertion (Fig. 3b). Our analysis suggests that rDNA units encoding this variant are clustered (Fig. 2). We anticipated that this clustering can be visualized in plant cells. We designed a locked nucleic acid (LNA) probe (CAT+), which specifically detects the CAT insertion located within NOR2. We performed a FISH experiment on spreads of pollen mother cells also employing an LNA probe directed against a universal rDNA sequence (SalI probe), detecting all 45S rDNA in *Arabidopsis*^18^ (Fig. 4a). We observed a single dominant CAT+ signal on NOR2 colocalizing with the universal SalI probe in wild-type plants (ecotype Col-0), strongly supporting the results of our sequencing efforts. We also investigated mutant plants with known instability of the NORs: *rtel1-1*, *nuc2-2*, and *hda6-6* mutants^18–20^. In the *rtel1-1*, *nuc2-2*, and *hda6-6* mutants, we detected a different localization of the CAT+ probe. The *hda6-6* and *rtel1-1* show a redistribution of the CAT cluster to both NORs, indicating a translocation event, whereas in the *nuc2-2* the CAT+ probe is much more prominent and covers a much larger area than the control.

We also developed a specific quantitative PCR (qPCR) assay that allows quantification of rDNA units containing the CAT sequence and puts them in relation to the total amount of rDNA units. According to the qPCR quantification, 9% of all rDNA units contain the CAT sequence in wild-type (Col-0) plants. This is in line with the 9% AF for the CAT sequence feature that we established by Illumina sequencing (Fig. 4b). We also quantified the rDNA units containing the CAT sequence in the mutant lines with NOR instability. We detect a two-fold increase of rDNA containing the CAT sequence in the *hda6-6*, *nuc2-2* mutant lines, which confirms the results obtained by the in situ hybridization experiments (Fig. 4b). In contrast, the *rtel1-1* mutant line shows no significant difference in copy number of the CAT, although cytologically the CAT is equally distributed between the NORs. Our results confirm the presence of the rDNA-CAT sub-cluster on NOR2 and provide a tool to visualize and analyze genomic rearrangements events between the NORs.

### Tissue-specific expression and ribosomal integration of rRNA variants

The generation of the first map of NOR2 and its information regarding sequence VARs within the rDNA units provide the possibility to attribute rRNAs to specific NORs and rDNA units. In this sense, we could assess whether different tissues preferentially express rDNA unit types which contribute to a heterogeneous ribosome population.

**Fig. 5:**
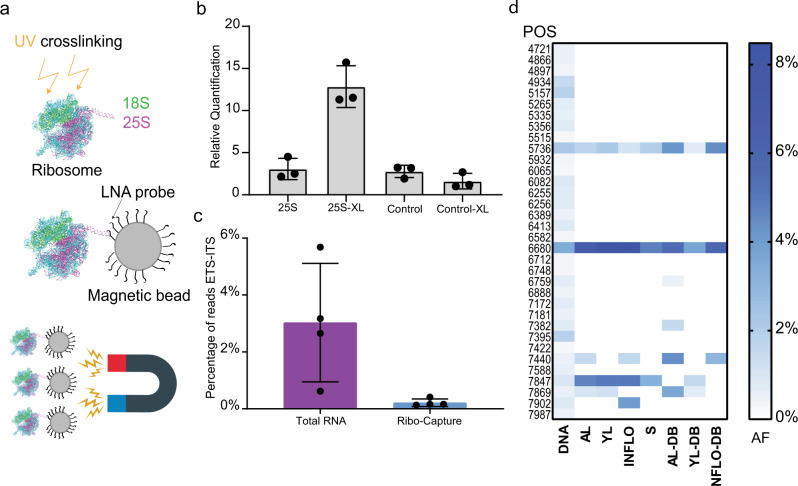
rRNA variants are differentially expressed between tissues. **a** Ribosome capture strategy for enriching mature ribosomes. Ribosomes are UV-crosslinked and captured with a magnetic bead coupled to a DNA/LNA mixamer with high affinity for the 25 S rRNA. **b** Graph depicting the relative quantification of 25S rRNA by qPCR after ribosome capture. Non-crosslinked ribosomes captured with the 25S LNA (25S), crosslinked ribosomes captured with the 25S probe (25S-XL), non-crosslinked ribosomes captured with an LNA probe that does not bind to the ribosome (Control), and crosslinked ribosomes captured with an LNA probe that does not bind to the ribosome (Control-XL). **c** Graph depicting the percentage of Illumina reads mapping to the ETS-ITS regions of the rDNA relative to the total amount of reads that map to the entire rDNA unit from total RNA and ribo-capture experiments. The reads from all tissues analyzed were used for this analysis. The ETS-ITS regions of the rDNA are present in the pre-rRNA but not present in mature ribosomes. **d** Heat map representing the allele frequency of SNPs found in the 25S of mature ribosomes in different tissue types. Blue boxes represent the highest value (8%), white boxes represent the lowest value (0%). The *y*-axis represents the positions of the SNPs with respect to the reference rDNA and are as follows: from top to bottom 4721, 4866, 4897, 4934, 5157, 5265, 5335, 5356, 5515, 5736, 5932, 6065, 6082, 6255, 6256, 6389, 6413, 6582, 6680, 6712, 6748, 6759, 6888, 7172, 7181, 7382, 7395, 7422, 7440, 7588, 7847, 7869, 7902, and 7987. The *x*-axis defines the different tissue types and sources: adult leaves (ALs), young leaves (YLs), inflorescences (INFLOs), siliques (S), online repository derived reads (DB), DNA (whole-genome sequencing data set).

Multiple SNPs and InDels were located in the 18S and 25S rRNA genes. To analyze whether these rRNA variants are transcribed and integrated into ribosomes, we sequenced total RNA and rRNA from different tissues: adult leaves (ALs), young leaves (YLs), inflorescences (INFLOs), and siliques (S). To enrich for rRNAs incorporated into ribosomes, we ultraviolet (UV)-crosslinked plant material and subsequently captured ribosomes with magnetic beads coupled to an LNA probe directed against the 25S rRNA. The enrichment of rRNA was validated by real-time qPCR (tissue from ALs) comparing non-crosslinked and crosslinked plant material incubated with the 25S LNA probe with an unrelated control LNA probe. We find an eightfold enrichment of rRNA in the crosslinked sample incubated with the 25S rRNA LNA probe, compared to the control samples (Fig. 5a, b).

The crosslinked samples, derived from four different tissues, enriched for rRNAs were further processed and sequenced (Illumina short-read 150 bp paired-end sequencing). As a comparison, total RNA of all tissues was also sequenced in the same manner. We evaluated sequencing reads from all ribosomal-enriched and all total RNA samples that mapped to the rRNA external and internal transcribed spacer (ETS/ITS) regions in relation to the total amount of reads mapping to the reference rDNA. The ETS/ITS regions are absent in the rRNAs integrated in the mature ribosomes but present in the pre-rRNAs^4^. We find a tenfold lower relative enrichment of ETS/ITS reads from ribosomal-enriched material compared to total RNA, indicating that the ribosomal enrichment strategy has been successful (Fig. 5c).

To identify whether there are tissue-specific rRNA variants, we mapped reads obtained from the rRNA samples (crosslinked, 25S rRNA LNA probe) to the reference rDNA, filtered the reads for quality (*q* > 30), and called SNPs and InDels with the software LoFreq. Only VARs found both in the rRNA and in the rDNA that are of the same quality were taken into consideration (Supplementary Table 2). Other sequence VARs present in the rRNA reads which originate from secondary modifications of the rRNA were omitted from the analysis. 22% of the SNPs/InDels found in the 25S rDNA are present within the 25S rRNAs and incorporated in ribosomes (Fig. 5d and Supplementary Table 2). Reads corresponding to the two SNPs at positions 5736 and 6680 within the 25S rRNA were found in all tissues but with different frequencies (Fig. 5d and Supplementary Table 2). It is interesting to note that, for instance, SNP 6680, which is a C to T transition, was found to have an AF of 3.4% when assessing the WG, yet a relative incidence of up to 8.4% when analyzing rRNA of mature ribosomes. Similarly, for several SNPs with low AF in the WG, corresponding reads of rRNAs were found with significantly higher frequency in mature ribosomes: SNP at position 7440 (WG = 0.5% AF) is expressed in ALs (1.4% AF) and INFLOs (1.6% AF); SNP at position 7869 (WG = 0.57% AF) is expressed in adult (1% AF) and YLs (1.2 % AF) (Fig. 5d and Supplementary Table 2). We could not detect reads corresponding to SNPs or InDels in the 18S rDNA in samples enriched for rRNA integrated into ribosomes.

To corroborate our findings, we analyzed previously published data sets of polysome profiling experiments performed in *A. thaliana* Col-0 plants using the same tissues indicated above^21–24^. Polysomes are the cellular fraction of ribosomes which are actively translating single mRNAs. Although the focus of these previous studies was mRNA, the data sets contained a large number of rRNA-related reads that we mapped to the reference rDNA^21–24^. More than 60% of the SNPs identified in our ribosome capture assays where also found in the polysome data sets (Supplementary Table 2 and Fig. 5d).

This set of experiments confirms that the rRNA gene variants we detect in the rDNA are expressed in a tissue-specific manner and present in translating ribosomes. Furthermore, the presence of only 25S rRNA variants suggests that NOR4 is mainly contributing to the pool of ribosomes even within tissues that have both NOR2 and NOR4 transcriptionally active (YLs and INFLOs)^6,18^.

## Discussion

We have generated a comprehensive assembly of the *A. thaliana* nucleolus organizer region of chromosome 2 by using a BAC-based approach^13^. Embedded in our study, we provide a sequencing and bioinformatic pipeline with ad hoc generated programs to aid the assembly of large and complex repetitive regions and address their higher-order organization. We followed a BAC-based approach because of the low complexity and the repetitive nature of this chromosomal region and to discriminate between rDNA units from NOR2 and NOR4. So far, attempts to reconstruct the NORs from genome-wide sequencing data yielded only very short rDNA contigs, which cannot be discriminated from assembly artefacts. The BAC-based strategy allowed us to build contigs with multiple rDNA repeats with confidence. The advantages of the BACs are manifold: the presence of a fixed and measurable number of rDNA units, the ease to obtain high-quality and long DNA molecules in large quantities, the potential to sequence the same BAC, and its rDNA units with two different methods, combining their advantages (read length via Nanopore; fidelity via Illumina).

The analysis of the NOR2 contigs allowed us to draw conclusions on the overall higher-order organization of the rDNA units and their heterogeneity. Our data show that the rDNA units are heterogeneous with many large and small VARs present in the internal and external transcribed spacer regions and, to some extent, in the core rRNA subunits. Although some of the sequence variants have already been identified before^7,9^, our data puts all of them in context of large sequence contigs. Our draft assembly is sufficiently complete to demonstrate that there are specific rDNA clusters that share similar sequence features, which could have originated from former rDNA homogenization events^3^. These events have led to the formation of large regions that contain rDNA units of the same type (100 kb, 10 rDNA units). Furthermore, our data allows attribution of processed rRNAs to their origin from chromosome 2 or 4 (Supplementary Data 4). Previously, only rDNA and unprocessed rRNA could be distinguished and attributed according to few polymorphism and their 3′-ETS variant^6,8,25^.

Based on a comparative analysis of short-read sequencing experiments performed on BACs (exclusively from NOR2) and genomic DNA, we were in the position to determine which SNPs/InDels are enriched on NOR2 and which on NOR4. Most of the unique sequence variants appear to be present on NOR4 or shared between NOR2 and NOR4.

The presence of rDNA clusters is supported by the analysis of spread meiotic chromosomes. Utilizing an LNA probe specifically directed against the CAT insertion rDNA unit type we detected signals only on NOR2. In addition, the probe allowed us to visualize rDNA expansion and re-arrangement events in the *hda6-6*, *nuc2-2* and *rtel1-1* mutant lines, which are known to have unstable rDNA copy numbers^18,19^. The expansions were further corroborated by qPCR experiments. We herewith generated the information and the tools to analyze (meiotic) recombination within and between the repetitive and highly similar rDNA domains by FISH.

As the NORs are conserved in their chromosomal structure and function among many organisms, it is plausible that they also have a similar cluster organization. It has been shown, in mice^26^ and bacteria^27^ that only a subset of rDNA repeats are transcribed at a given time. This would indicate a cluster-like organization and regulation of the rDNA units.

Interestingly, only up to 20% of the SNPs contained within the 25S rDNA were detected in mature ribosomes. The two SNPs at positions 5736 and 6680, which are expressed in all tissues analyzed, are located within the ribosomal extension segment (ES) 12 and the ES27 of the large ribosomal subunit, which are known to be highly variable regions^26^. In contrast, the SNP at position 7440, which is expressed only in ALs and INFLOs, is located on the loop between H74 and H88, which is located in the ribosome peptidyl transferase site^28^. This SNP is a G to T transition and could have a functional impact on the ribosome by changing the interaction of the tRNA with the ribosome.

Our data implies that only a subset of the rRNA genes is transcribed at a given time in a well-controlled manner. Furthermore, none of the larger deletions or insertions that were detected in rDNA units (Supplementary Fig. 3b, c) are detected in the total RNA data sets or in the mature ribosomes. This could indicate that these large deletions perturb the maturation of the rRNA into fully folded ribosomes. These results support the findings in mice^26^ and bacteria^27^, where ribosomal variants are tissue specifically expressed or enriched under certain stress conditions. Together with our results, the presence of rRNA variants subpopulations across different organisms reinforces the hypothesis of specialized ribosomes and demonstrates the fine-tuned transcriptional regulation of the rDNA repeats.

It is interesting to note that we could never detect rRNAs bearing the CAT insertion in any of our and previously published^21–24^ total RNA-sequencing data sets. This indicates that even though both NORs are transcriptionally active in young seedlings^29^ and in INFLOs^18^, not all repeats of NOR2 are transcribed. This is in contrast to what was previously hypothesized, as NOR2 is partially transcriptionally active in seedlings. These results reveal a complex, multi-level regulation of rRNA gene transcription and rRNA integration. The locus-specific epigenetic silencing of NOR2, as a whole, was previously shown only on the base of the expression of the 3′-ETS^9^. In contrast, our data sets, with the precise positioning of each rDNA unit to a defined cluster, suggest that rDNA silencing might be confined to the clusters themselves. We conclude that clusters of rRNA unit types are co-regulated in a tissue-specific manner supporting the concept of tissue-specific ribosome subpopulations differing in their rRNA composition.

## Methods

### BAC library

The BAC library used in this study was established by Mozo et al.^15^ at the “Institut für Genbiologische Forschung” in Berlin, Germany. The library is available at the ABRC (https://abrc.osu.edu/).

### BAC extraction

BAC DNA extraction has been carried out according to the “Qiagen Large Construct extraction protocol” (April 2012), which has been specifically designed to retrieve large amounts of long, circular and intact DNA, such as BACs and Cosmids.

A bacterial culture or a single colony was inoculated in 500 ml of Loading Beads (LB) liquid media containing the appropriate selective antibiotic (kanamycin, final concentration 100 µg/ml) and grown overnight at 37 °C with vigorous shaking (300 r.p.m.). Cells were collected through centrifugation (6000 × *g*) for 15 min at 4 °C. Here, 6000 × *g* corresponds to 6000 r.p.m. in Sorvall GSA or GS3 of Beckman JA-10 rotors. The pellet was resuspended in 20 ml of Buffer P1 (Resuspension Buffer) containing RNaseA (with final concentration 100 µg/ml). The cells were lysed by adding 20 ml of Buffer P2 (Lysis Buffer). pH equilibration was achieved by adding 20 ml of Buffer P3 (Neutralization Buffer). After centrifugation at >20,000 × *g* for 30 min at 4 °C (a centrifugal force of 20,000 × *g* corresponds to 12,000 r.p.m. in a Becman JA-17 rotor or 13,000 r.p.m. in a Sorvall SS-34 rotor), the supernatant containing the BAC DNA was transferred to a new centrifugation tube. A cell strainer (70 µm) was used to separate effectively cell debris from the supernatant. The DNA was precipitated by adding 35 ml of isopropanol at room temperature and collected by centrifugation (>15,000 × *g* for 30 min at 4 °C) and washed with 5 ml of ethanol (70%). After removal of alcohol, the pellet was air-dried for 10 min.

To select for circular and unfragmented DNA, an exonuclease digestion step was performed (NEB T5 exonuclease, 10,000 unit/ml). Reaction buffer (9.5 ml) was added to the dried pellet for resuspension, followed by addition of 500 µl of EX buffer and 1 µl of exonuclease and incubation at 37 °C for 1 h. In the meantime, the filtration columns were equilibrated (QIAGEN-tip 100) with 15 ml of buffer QBT (Equilibration Buffer). Then, 10 ml of Buffer QS (Adjustment Buffer) were added to the sample and the sample was thereafter loaded onto the column. After the DNA has entered the resin, 60 ml of buffer QC (Wash buffer) were added. The BAC DNA was eluted by adding 15 ml of Buffer QF (Elution Buffer).

The DNA was precipitated by the adding isopropanol (as above) and then washed once with 70% ethanol. After having removed the ethanol, the pellet was air-dried for 20 min and then resuspend in 100 µl of Tris-EDTA (TE) buffer. To enhance DNA resuspension, TE buffer was pre-warmed to 65 °C.

### BAC linearization

BACs extracted from *Escherichia coli* were linearized with the restriction enzyme ApaI (NEB). This enzyme was selected, as it is a single cutter in the vector backbone (pBeloBAC-Kan) and according to available sequence information leaves the rDNA repeats intact. One microliter of enzyme (50 units) was added to 1 µg of BAC DNA. The reaction was incubated overnight at 37 °C. Enzyme inactivation was carried out by incubating the sample at 65 °C for 20 min.

### Pulse-field gel electrophoresis

The successful linearization of the BACs has been assessed through PFGE using the Clamped Homogeneous Electric Fields technology. Two percent low-melting agarose solution (Certified^TM^ Low Melt Agarose, BioRad) was prepared using 1× TBE buffer (Tris base 1 M, Boric Acid 1 M, EDTA 0.02 M) and melted using a microwave. The solution was equilibrated at 50 °C in a water bath. One microgram of linearized BAC DNA was combined with equal volume of 2% Certified^TM^ Low Melt Agarose and mixed by pipetting, to achieve a final concentration of 1% agarose. The mixture was quickly transferred to plug molds and left to solidify for 10 min. While waiting for the solidification of the plugs, 1% agarose gel (Pulse Field Certified Agarose, BioRad) was prepared using 1× TBE buffer and casted in a PFGE casting stand. Finally, the plugs were inserted inside the wells. PFGE was run according to the following program for 20 h: switch time 200 s, reorientation angle 120 °C, voltage gradient 5 V/cm. The final result was visualized through a transilluminator by staining the gel with EtBr for 1 h in 1× TBE.

### rDNA-CAT copy number quantification

DNA was extracted by crushing leaves in UREA buffer (0.3 M NaCl, 30 mM TRIS-Cl pH 8, 20 mM EDTA pH 8, 1% [w/v] *N*-lauroylsarcosine, 7 M urea) and subsequently purified with phenol : chloroform : isoamylalcohol (25 : 24 : 1). The qPCR reaction was performed using the KAPA SYBR FAST kit following the product specifications. To quantify rDNA-CAT copy numbers, 20–30 ng of genomic DNA were used together with two primer pair sets: 18SRealdn and 18SRealup as previously described for *Arabidopsis*^30^ to amplify all rDNA copies, CAT+ fw (5′-CGC ATC AGC AAA GGA TGA TGG-3′)–CAT+ rv (5′-AGT CTA AAA CGA CTC TCG GCA-3′) to amplify only the rDNA-CAT. As a Ct calibrator for the experiment, BAC F2J3 was used, as it has nine rDNA copies of which one with the CAT insertion. In addition, for each genomic sample, the reference gene *ACTIN-7* was used to calculate the rDNA copy number. The amplification of the rDNA, rDNA-CAT, and of the reference gene *ACTIN-7* was performed in separate wells and in three technical replicates. The analysis was conducted with two separate biological replicates. The conditions used for the qPCR were 95 °C 1 min initial denaturation, 95 °C 30 s, 65 °C 30 s, 72 °C 30 s for 40 cycles with fluorescence detection after every elongation step. The PCR products were not longer than 250 bp and contained a GC content of ∼50%. The experiment was performed on an Eppendorf Realplex 2 Mastercycler.

### 3′-ETS variant PCR

Amplification of the 3′-ETS variants was performed as in Pontvianne et al.^18,31^. In brief, a PCR reaction was performed on extracted BACs with the following primers “3allrRNAVAR” (5′-CTGGTCGAGGAATCCTGGACGATT-3′) and “5allrRNAVAR” (5′-GACAGACTTGTCCAAAACGCCCACC-3′). The PCR was run with the following program: 95 °C for 2 minutes for initial denaturation, 95 °C for 30 s–55 °C for 30 s–72 °C for 30 s were repeated for 40 cycles, final elongation was set at 72 °C for 2 min.

### Westburg preparation for Illumina sequencing

BACs were prepared for Illumina sequencing according to the “Westburg NGS DNA library prep kit” (protocol 3.1 10/2018) with minor optimizations. First, the DNA was fragmented through an enzyme mix provided in the kit. Five microliters of fragmentation mix (10×) are added to 100 ng of DNA (final volume of the reaction: 40 µl) and left for 30 min at 65 °C, after an equilibration period at 32 °C for 20 min.

Second, the DNA fragments are linked to the sequencing adaptors and barcoded through PCR. Illumina adaptor are mixed together equimolar amounts of forward and reverse oligos to obtain a final concentration of 1.5 µM. The mix was incubated at 95 °C for 5 min on a thermocycler and left to cool down for 30 min. Annealed Illumina adapter (2.5 µl) is mixed with 40 µl of the fragmented DNA together with 17.5 µl of H_2_O, 20 µl 5× ligation buffer, and 10 µl of DNA ligase. Samples were incubated at room temperature for 15 min. Eighty microliters of AmpureXP beads were added and mixed by pipetting or vortexing. Captured magnetic beads are washed once with 85 % EtOH and resuspended in 50 µl of water. Twenty microliters of Binding solution are added (20% PEG8000, 2.5 M NaCl, 0.05% Tween 20), mixed thoroughly, and incubated 5 min at room temperature. After capturing the beads, the supernatant is transferred to a fresh well and 20 µl of new beads added. The samples are incubated for 5 min at room temperature. The beads were washed twice with 85% ethanol and left to dry for 1 min. The samples were suspended in 11 µl of water for 2 min at room temperature. After capturing the beads, 10 µl of the supernatant were transferred to a fresh well. Fifteen microliters of 2× KAPA HF Ready Mix were added with 2 µl of the appropriate barcode, 2 µl of the TrueSeq Universal Adapter, and 1 µl of H_2_O. The sampels were ran with the following PCR program: 98 °C 1 min, 98 °C 5 s–65 °C 30 s–72 °C 1 min for 15 times, 72 °C 1 min.

Finally, the samples were purified and cleaned by AmpureXP beads. Twenty-seven microliters of AmpureXP beads were mixed by pipetting or vortexing with the sample. The beads were captured and the supernatant was discarded; the pellet was washed twice with 85% ethanol. The beads were resuspended in 25 µl of water, the beads were captured and the supernatant transferred to a fresh well. The samples were now submitted for sequencing.

### Nanopore library preparation with native barcoding

Linearized BACs have been sequenced through Oxford Nanopore Technology. Twelve linearized BACs were multiplexed, exception made for BACs F2J17 and F1E12, which were processed individually (Supplementary Table 1) and sequenced with a Nanopore MinION using an R9.4 flow cell (FLO-MIN109, ONT). For each BAC, only ultra-long reads (longer than 50 kb) were used for the assembly, exception made for BAC F1L21 (Supplementary Fig. 1c and Supplementary Table 1). Each BAC was assembled by using the software CANU^32^ and polished three times using the software Nanopolish^33^.

Multiplexing preparation followed the manufacture’s manual “1D Native barcoding genomic DNA -SQK-LSK109” (version: NBE_9065_v109_revH_23May2018) with some optimizations.

### DNA repair and end-prep

In the first step, DNA repair was achieved through the utilization of a cocktail of enzymes (NEBNext FFPE DNA repair Mix) designed specifically to repair breaks in the DNA. This cocktail is able to repair nicks, gaps (up to ten nucleotides), blocked 3′-ends, oxidized bases, and deamination of cytosine to uracil. Afterwards, the DNA ends are repaired through the Ultra II End-prep enzyme mix, which enhances the attachment of the DNA barcodes. It is important to note that BACs are extremely sensible to physical sharing. For this reason, throughout this protocol, we mixed the samples always by manual flicking to avoid to pipette and vortex. DNA (1.2–1.5 µg) were transferred into a 1.5 ml DNA LoBind tube and the volume adjusted to 48 µl with Nuclease-free water and mixed thoroughly by inversion or flicking. We added to the DNA following exactly this order directly in the LoBind tube, 3.5 µl NEBNext FFPE DNA Repair Buffer, 2 µl NEBNext FFPE DNA Repair Mix, 3.5 µl Ultra II End-prep reaction buffer, and 3 µl Ultra II End-prep enzyme mix. The samples were incubated at 20 °C for 5 min and 65 °C for 5 min. During the incubation we prepared 500 µl of fresh 80 % ethanol in Nuclease-free water. We added 60 µl of resuspended AMPureXP beads to the end-prep reaction and mixed by flicking the tube. The samples were incubated on a Hula mixer (rotator mix) for 15 min at room temperature, then spun down briefly and placed on a magnetic rack. We kept the LoBind tube on the rack and pipetted off the supernatant, then the beads were washed twice with 200 µl of 80% ethanol. The samples were spanned down and placed back on the magnet. The residual ethanol was removed and the samples left to air dry for 60 s. The samples were resuspend by flicking in 25 µl Nuclease-free water. To enhance elution, we incubated the samples for 20 min at 37 °C by using a thermocycler.

The pellet was spun down and the sample placed on a magnetic rack until the eluate is clear and colorless, we removed and retained 25 µl of eluate into a clean 1.5 Eppendor DNA LoBind tube.

One microliter of end-prepped DNA was measured using a Qubit fluorometer (recovery aim > 200 ng).

We then proceeded with the naive barcoding step.

### Barcode ligation

The second step consists on barcoding the samples to sequence them together. We added to the 24 µl of repaired and end-prepped DNA, 2.5 µl Native Barcode (Native Barcode 1-12, EXP-NBD104), and 25 µl Blunt/TA Ligase Master Mix, maintaining this order. We mixed by flicking and briefly spanned down. The samples were incubated at room temperature for 10 min. We added 50 µl of resuspended AMPureXP beads to the reaction and mixed by flicking, then incubated the sample on a rotator mix for 15 mi at room temperature. The sample was spanned down and the sample placed on a magnetic rack; the supernatant was removed and the beads washed twice with 80% ethanol. Residual ethanol was removed and the sample left to air dry for 60 s (do not dry the pellet to the point of cracking). The tube was removed from the magnetic rack and resuspended by flicking in 26 µl Nuclease-free water. To enhance elution, the sample was incubated for 20 min at 37 °C.

The sample was placed on a magnetic rack until the eluate was clear and colorless. We then removed and retained 26 µl of eluate containing the DNA library into a clean 1.5 Eppendor DNA LoBind tube.

The barcoded samples were pooled together into a 1.5 ml Eppendorf DNA LoBind tube, ensuring that sufficient sample is combined to produce a pooled sample of >400 ng total. It is not fundamental to pool equimolar amounts of the samples, as long as the concentrations are not too different (differences in the final quantity of barcoded samples between 50 and 80 ng are acceptable). If the volume of the pooled samples exceeded 65 µl, it was necessary to perform an additional concentration step.

Pooled barcoded samples were quantified using a Qubit fluorometer (recovery aim > 500 ng).

### Adapter ligation and clean-up

The final step consists on the ligation of the adapters needed for sequencing to the DNA fragments. This is achieved by adding the proprietary Nanopore adaptor (Adapter Mix II) with the barcoded DNA and the T4 DNA ligase. Adapter ligation was performed by mixing by flicking the tube between each sequential addition of: 65 µl pooled barcoded sample, 5 µl Adapter Mix (AMII), 20 µl NEBNext Quick Ligation Reaction Buffer (5×), and 10 µl Quick T4 DNA Ligase.

The samples were mixed by flicking incubated for 10 min at room temperature. Fifty microliters of resuspended AMPureXP beads were added to the reaction and mixed by flicking, then incubated on a rotator mixer for 15 min at room temperature. The samples were placed on a magnetic rack and the beads allowed to pellet. The supernatant was removed and the beads washed twice by adding 250 µl Long Fragment Buffer and left to dry for 30 s. The sample was resuspended in 15 µl Elution Buffer and incubated for 20 min at 37 °C. The samples was placed on a magnetic rack until the eluate is clear and colorless. The supernatant was removed and retained into a clean 1.5 ml Eppendorf DNA LoBind tube. The library was now ready to be sequenced.

### Priming and loading the SpotON flow cell

In this step the flow cell is put under pressure and prepared for sequencing by the addition of the appropriate buffers. Pressurization enhances the correct flowing of the buffers in the device. The sample were mixed with the LB that take the DNA molecules close to the pores.

The flow cell was put under pressure by opening only the priming port, a P1000 pipette was set to 200 µl, inserted the tip into the priming port, and turned the wheel until the dial shows 200–230 µl, or until it was possible to see a small volume of buffer entering the pipette tip. The flow cell priming mix was prepared by adding 30 µl of thawed and mixed Flush Tether directly to the tube of thawed and mixed Flush Buffer, and mixed by pipetting up and down. Eight hundred microliters of the priming mix were loaded into the flow cell via the priming port, avoiding air bubbles.

The final library was prepared for sequencing by mixing together 37.5 µl Sequencing Buffer (SQB) with 25.5 µl LB (taking care to mix them immediately before use, as they precipitate quickly) and 14 µl of the DNA library. The prepared library was mixed by gently pipetting up and down just prior to loading. We completed the flow cell priming by gently lifting the SpotON sample port cover to make the SpotON sample port accessible, then by loading 200 µl of the priming mix into the flow cell via the priming port, avoiding the introduction of air bubbles. The prepared DNA library was mixed gently and loaded to the flow cell via the SpotON sample port in a dropwise fashion. The sequencing runs were carried out until all pores were completely exhausted and generated on average 7.12 Gb of raw sequence information.

### *A. thaliana* growth conditions

*A. thaliana* Col-0 ecotype seeds were stratified in water in the dark at 4 °C for 2 days before sowing on soil/perlite 3 : 1 mixture (ED 63, Premium Perlite). Pots were covered with a transparent lid until cotyledons were fully developed and first primary leaves visible. Plants were grown under long day conditions in controlled environment rooms (16 h of light, 8 h of darkness, 60–80% humidity, 21 °C, 15,550 lux, T5 Tube illumination).

### DAPI spreads

INFLOs were harvested into fresh fixative (3 : 1 96% [v/v] ethanol [Merck] and glacial acetic acid) and kept overnight (O/N) for fixation. Once the fixative decolorized the INFLOs, they were placed in fresh fixative (can be stored for over a month at −20 °C), and subsequently, one INFLOs was transferred to a watch glass. The yellow buds were removed to collect only white and transparent buds. The white buds were separated from the INFLOs and grouped according to size. This step is necessary to obtain preparations with separated meiotic stages.

Afterwards, the buds were washed three times with citrate buffer (0.455 mL of 0.1 M citric acid, 0.555 mL of 0.1 M trisodium citrate in 10 mL of distilled water) and submerged in an enzyme mix (0.3% w/v cellulase, 0.3% w/v pectolyase in citrate buffer). Each bud has to be submerged for the digestion to work efficiently. The buds were incubated for 90 min in a moisture chamber at 37 °C. Digestion was inhibited by adding cold citrate buffer. At this point, the buds were transferred (maximum three to four buds of the same size) to a glass slide. Excess liquid was removed and 15 μl of 60% acetic acid added. The buds were suspended using a metal rod and an additional 10 μl of 60% acetic acid was added to the suspension. The droplet area was labeled using a diamond needle and fixed with fixative. Slides were dried for at least 2 h. To stage the meiocytes, 15 μl of 2 µg/ml DAPI (4’,6 diamidino-2-phenylindol) diluted in Vectashield (Vector Laboratories) was added to the slide and sealed with a glass cover slip. Images were taken on a Zeiss Axioplan microscope (Carl Zeiss) equipped with a mono cool-view charge-coupled device camera^34^.

### Fluorescence in situ hybridization

The slides selected for FISH were washed with 2× saline-sodium citrate buffer (SSC) for 10 min and incubated for 2 min at 37 °C in pre-heated 0.01 M HCl with 250 µl of 10 mg/ml of Pepsin and 5 µl of 100 mg/ml RNAseA. The slides were then washed in SCC for 10 min at room temperature. Fifteen microliters of 4% paraformaldehyde were added onto the slides, covered with a strip of autoclave bag, and placed for 10 min in the dark at room temperature. The slides were then washed with deionized water for 1 min and dehydrated by passing through an alcohol series of 70, 90, and 100%, for 2 min each. Slides were left to air dry for 30 min. The probe mix was prepared by diluting 1 μL of probe (50 pmol of LNA probe) in a total of 20 μL of hybridization mix (10% dextran sulfate 50,000 MW, 50% formamide in 2× SSC).

The probe mix was denatured at 95 °C for 10 min and then placed on ice for 5 min. Afterwards, the probe mix was added to the slide, covered with a glass cover slip, sealed, and placed on a hot plate for 4 min in the dark at 75 °C. Finally, the slides were placed in a humidity chamber overnight at 37 °C. After hybridization, the cover slips were carefully removed and the slides were treated with 50% formamide in 2× SCC for 5 min in the dark at 42 °C. The slides were then washed twice with 2× SCC for 5 min in the dark at room temperature. Slides were visualized on an inverted epi-fluorescent microscope equipped with an CCD camera. LNA probes used in this study are: ATG+ (5′-CCC TCA CCA TCA TCC TT-3′) labeled with TYE563 (red) and SalI^18^.

### CAPs marker confirmation

One microgram of DNA from BACs F2D9, F2G3, F2G18, F2E13, and F2I6 were tested for the presence or absence of the restriction site (AvaI) at position 4133 of the reference rDNA. The region harboring the restriction site was amplified by PCR (primer sequences: AVAI Fw 5′-CGCATCAGCAAAGGATGATGG-3′, AVAI Rv 5′-ACCTTGGGATGGGTCGG-3′). The final amplicons were digested with the enzyme AvaI. Seventeen microliters of the PCR reaction were mixed with 2 µl of CutSmart Buffer and 1 µl of AvaI (NEB) for a final volume of 20 µl. The reaction was incubated at 37 °C overnight. The samples were loaded on an agarose gel (1 % w/v + EtBr) and visualized on a Biorad transilluminator.

### Ribo-capture protocol

Ribosome captures were performed as in Rogell et al.^35^ with some modifications. Magnetic beads (Dynabeads M-270 Carboxylic Acid) were activated and coated with ad hoc designed LNA probes (Qiagen), harboring an -NH_2_ group, following the manufacturer’s instructions. The beads were stored for a maximum of 3 months. Ribosome capture was performed as explained in Rogell et al.^35^. In brief, plants were UV (260 nm) crosslinked in a Stratalinker at 0,15 J/cm^3^ and placed at 10 cm from the light source. After irradiation the plant material was immediately frozen in liquid nitrogen and stored at −80 °C. Plant material was ground in 1 ml Lysis Buffer (20 mM Tris-HCl pH 7.5, 500 mM LiCl, 0.5% LiDS [w/vol, stock 10%], 1 mM EDTA, and 5 mM dithiothreitol (DTT)) with the addition of 1 µl of Protease Inhibitor (Thermo Fisher) and 1 µl of RNAse inhibitor (RiboLock). Fifteen percent v/v of formamide or ethylene carbonate were added to the sample as a hybridization enhancer. The samples were placed at 60 °C for 5 min. Beads coated with the LNA probe were denaturated at 95 °C for 3 min and added to the sample. The mix was placed at 40 °C for 2 h shaking. Afterwards, the samples were placed in a magnetic rack and washed three times with Lysis Buffer, twice with Buffer 1 (20 mM Tris-HCl pH 7.5, 500 mM LiCl, 0.1% LiDS [wt/vol], 1 mM EDTA, and 5 mM DTT), Buffer 2 (20 mM Tris-HCl pH 7.5, 500 mM LiCl, 1 mM EDTA, and 5 mM DTT), and Buffer 3 (20 mM Tris-HCl pH 7.5, 200 mM LiCl, 1 mM EDTA, and 5 mM DTT). The captured RNA was eluted in 100 µl of RNAse free water for 3 min at 90 °C. rRNA was purified by a DNAseI (Thermo Fisher) clean up, Proteinase K clean up, and phenol : chloroform : isoamylalcohol (25 : 24 : 1) pH 5 extraction. At this step, the samples are ready for library preparation and reverse-transcription PCR (RT-PCR). The Lexogen CORALL RNA-seq library kit was used for preparing the samples for Illumina sequencing. cDNA for qPCR analysis was obtained by using the iSCRIPT kit following the manufacturer’s instructions. The qPCR reactions were performed using the KAPA SYBR FAST kit following the product specifications. 25 S directed LNA probe sequence: 5′-AACGCCGAAGACGTCCGAT-3′. Mock LNA probe sequence: 5′-AAGACGTCGAAGGTTACCT-3′.

### Total RNA sequencing

Plant material from different *Arabidopsis* tissues was collected and frozen immediately in liquid nitrogen. To extract RNA, the SV Total RNA Extraction Kit (Promega) was used following the product specifications. Total RNA was further purified by a DNAseI (thermofisher) clean up and phenol : chloroform : isoamylalcohol (25 : 24 : 1) pH 5 extraction. At this step, the samples are ready for library preparation and RT-PCR. Lexogen CORALL RNA-seq library kit was used for preparing the samples for Illumina sequencing.

### MinION sequencing

MinION sequencing was performed according to manufacturer’s guidelines using R9.4 flowcells (FLO-MIN109, ONT). The run was monitored using Oxford Nanopore Technologies MinKNOW software. The specific version of the software has changed from run to run and is included in the fast5 files generated. The base-calling has been conducted with Albacore (version 2.0) for BACs F23H14, F2J17, F1E12, and Guppy (version 2.2.3) for all the other BACs. A list of all Nanopore sequencing runs are described in Supplementary Table 4.

### Assembly

Assembly has been conducted with Canu (v1.7.1 and v1.8) starting from fastq files with reads longer than 50 kb. It is important to note that assemblies carried out by using reads with a Phred quality score > 9 required less reads than those with a low-quality score. The command used is the following for all the BACs:

canu overlapper = mhap utgReAlign = true -d /path/to/assembly_directory -p assembly_prefix -t 40 genomeSize = 0.1 m -nanopore-raw ont_reads.fastq

The MHAP algorithm was chosen because of its ability to handle repetitive elements. utgReAlign= true allows to compute a sequence alignment for each overlap produced by mhap. The final assemblies (draft_assembly.fasta) are outputted in the fasta format.

### Assembly polishing using Nanopolish

Assemblies generated by Canu have been refined using the nanopolish consensus-calling algorithm (v0.10.2 and v.0.11). Each assembly has been polished using all the reads contained in the original sequencing fastq file.

minimap2 (version 2.14) was used to map the reads to the draft assembly. The command and the options used were the following:

minimap2 -ax map-ont -t 8 draft_assembly.fasta ont_reads.fastq | samtools sort -o mapped_ont_reads.bam -T mapped_reads.tmp

Nanopolish was used to produce an improved consensus sequence for the draft BAC assembly produced by Canu. The command and the options used were the following:

nanopolish variants –-consensus -o polished_assembly.vcf -r ont_reads.fastq -b mapped_ont_reads.bam -g draft_assembly.fasta

nanopolish outputs a Variant Calling Format file (polished_assembly.vcf). To generate the polished genome in fasta format, it is necessary to run the following command:

nanopolish vcf2fasta -–skip-checks -g draft_assembly.fasta polished_assembly.vcf > final_assembly.fasta

### Evaluation of the assembly

The Illumina reads of each BAC were mapped with the software bowtie2^36^ to a reference rDNA repeat. SNPs and InDels were called with LoFreq^37^, a base quality–aware algorithm designed for conservative detection of rare sequence variants that implements a strand bias test with Bonferroni correction^37^. The correctness of the long-read assemblies was confirmed by: (i) restriction digest with FspI and XhoI, which releases the repetitive SalI boxes, a prominent length polymorphism marker in the rDNA^5^; (ii) CAPS analysis of a frequent polymorphism at position 4133 (position relates to reference rDNA), which is cut by the AvaI restriction enzyme^7^; and (iii) by comparing the nature and the numbers of SNPs and InDels in a given assembly with the SNP/InDel AFs derived by short-read Illumina sequencing.

### SNPs and InDels calling

SNPs and InDels present on each BAC have been identified through Illumina sequencing. Illumina reads were mapped to the reference ribosomal DNA repeat and SNPs/InDels calling was performed using LoFreq^37^. Reads were mapped using bowtie2^36^ and sorted with samtools^38^.

The quality score for SNPs/InDels was calculated with LoFreq:

lofreq indelqual –-dindel mapped&filetered_illumina_reads.sorted.bam -f reference_repeat.fasta > final_illumina_reads.bam

SNPs and InDels were called with the following command:

lofreq call -–call-InDels final_illumina_reads.bam -f reference_repeat.fasta > called_variations.vcf

The same procedure was used for calling the SNPs/InDels in the WG samples (Kurzbauer et al. (2020), submitted) and the ribo-capture samples.

### rDNA reference repeat

The rDNA reference repeat (Supplementary Data 5) was generated starting from one rDNA gene of BAC F2J17 (variant 1). The repeat was polished by Pilon (five rounds) using WG *A. thaliana* Illumina reads (Kurzbauer et al. (2020), submitted, PRJNA555773). The SalI boxes were removed from the reference.

### OverBACer (overlap BAC finder)

To identify the overlaps between BAC assemblies, a python script was devised named OverBACer. OverBACer exploits the different lengths of SalI boxes, 3′-ETS variants and other large InDels occurring within the rDNA repeats, to find the overlapping genes between two or more BACs.

OverBACer takes as input a file containing the assemblies to be analyzed (in fasta format), a search file consisting of the probes to be identified in the assemblies (in fasta format), and the maximum/minimum number of repeats needed to call an overlap.

The algorithm is divided into three steps: reference point identification, distance calculation between such points and overlap.

In the first step, OverBACer identifies the position in the assemblies of the given probes present in the search file. It accomplishes this task by performing a “word search” within the assembly. Every time a probe finds a match on the sequence, its absolute position on the assembly is annotated; these positions are called “reference points”.

In the distance calculation step, OverBACer computes the distances between neighboring reference points. This system allows to “barcode” the sequence, creating a fingerprint of each assembly. As each rDNA repeat has a slightly different SalI box length, the distances between two reference points will vary among repeats, allowing to distinguish different rDNA genes. Moreover, the combination of the distances of each repeat within an assembly assigns a unique tag to each BAC.

In the overlap step, the barcodes of each assembly are compared with all the others, thus finding the overlaps. This task is performed by checking the correct order of the reference points on the sequences and the distances associated to the reference points. In particular, a tolerance chosen by the user is applied when comparing the distances, thus taking into account the presence on the sequence of small VARs such as SNPs and small InDels that might prevent the identification of correct overlaps.

Usage example:

OverBACer –i assemblies.fasta –o output_file.txt –s search_probe_file.fasta –max_n 30 –min_n 3 –t 13

Where –i specifies the input file containing the assemblies to be overlapped, –o the output file, –s the search file consisting on the probes to be used to identify the reference points, –max_n and –min_n the maximum/minimum number of reference points to call an overlap, and –t the tolerance applied when the distances are compared (in bp). The program was calibrated to find a minimum of two overlapping repeats with an error range of ±35 bp.

### Neighbor Finder

To study the association of SalI boxes and small SNP/InDels of rDNA repeats at 10, 20, and 30 kb, a python script (Neighbor Finder) was designed.

Neighbor Finder takes as input a text file consisting of the barcoded BACs classified through the alphanumerical system As explained in the results section. It analyzes the co-occurrence SalI boxes (-a 0 option) and of small SNP/InDels (-a 1 option). For all pairwise combinations of barcodes, we count how often a pair of barcodes occurs at 10, 20, and 30 kb distances. Figure 3d–f show the results. As an example, the association of SalI boxes is described below. In both cases, it is possible to randomize the data by using the -r 1 option.

Neighbor Finder extracts from the barcodes the portion of the alphanumeric code that describes the SalI box length, which is represented by the letters between round brackets. Then, the code of two neighboring repeats (at 10, 20, or 30 kb of distance) is fused to give a combination of SalI boxes, which is then searched within the pool of all the combined codes generated for each BAC. Neighbor Finder counts the occurrence of each combined code within the pool and returns this number associated with the corresponding code. The process is repeated three times in order to evaluate the association of SalI boxes at 10, 20, and 30 kb.

Usage example:

neighbor_finder -i input_file -o output_file -t temporary file -a 0 -r 0

Where -i specifies the input file (BACs have to be classified using the alphanumerical system, with the SalI boxes of each repeat in brackets and each repeat separated by a tab), -o the output file, -t the temporary file, -a the type of analysis (0 for SalI boxes and 1 for SNPs/InDels), and -r for the randomization (0 = no randomization, 1 = randomization). The normalized association values were obtained by dividing the number of each rDNA association by the sum of the number of repeats taken into consideration. A list of all BACs with each rDNA unit categorized by their features is described in Supplementary Data 6.

### Monte Carlo simulation

The Monte Carlo simulation was performed running the Neighbor Finder with an initial adaptation to shuffle the rDNA units within and between the BACs for 1000 times. The frequency of each rDNA association was calculated by summing the number of occurrences per association divided by 1000. We considered only frequencies larger than 0.90. The normalized association values were obtained by dividing the frequency of each rDNA association by the sum of the number of repeats taken into consideration.

### Statistical analysis

All statistical analyses, *t*-tests, Mann–Whitney test, and Pearson’s correlation tests (as indicated in the figure legends) were performed using GraphPad Prism 8 software (GraphPad Software). Unpaired, two-tailed Mann–Whitney tests were performed, as D’Agostino Pearson omnibus K2 normality testing revealed that most data were not sampled from a Gaussian population, and nonparametric tests were therefore required. Error bars indicate SDs.

### Preprint

A previous version of this manuscript was published as a preprint[x].

### Reporting summary

Further information on research design is available in the Nature Research Reporting Summary linked to this article.

## Supplementary information

Supplementary Information

Peer Review

Description of Additional Supplementary Files

Supplementary Dataset 1

Supplementary Dataset 2

Supplementary Dataset 3

Supplementary Dataset 4

Supplementary Dataset 5

Supplementary Dataset 6

Reporting Summary

## Data Availability

Accession numbers for all BACs are listed in Supplementary Table 3. All sequencing data is deposited on the Sequencing Read Archive (SRA) under BioProject number PRJNA632576. The whole-genome sequencing data set from Kurzbauer et al. (2020) is deposited under BioProject number PRJNA555773. Raw data sets used for Figs. 2 and 4, and Supplementary Fig. 4 are listed in the Supplementary Information. Source data are provided with this paper.

## Source data

Source Data

## Code availability

Custom codes used in this study, NeighbourFinder [10.5281/zenodo.4320729]^39^ and OverBACer [10.5281/zenodo.4320680]^40^, were deposited on Github (https://github.com/giovannisestini/neighbour_finder)(https://github.com/giovannisestini/overBACer).
