# Supplementary material for: Sequencing of the Arabidopsis NOR2 reveals its distinct organization and tissue-specific rRNA ribosomal variants
Source: Nat Commun. 2021 Jan 15;12:387. doi: 10.1038/s41467-020-20728-6 (PMC7810690; doi:10.1038/s41467-020-20728-6)
Supplement: Supplementary file 1 — Supplementary Information [file 41467_2020_20728_MOESM1_ESM.pdf]

Sequencing of the *Arabidopsis* NOR2 reveals its distinct organization and tissue-specific rRNA ribosomal variants

Jason Sims[1,3,#], Giovanni Sestini[1,3,4], Christiane Elgert[2], Arndt von Haeseler[2] and Peter Schlögelhofer[1,#]

[1]Department of Chromosome Biology, Max Perutz Labs, University of Vienna, Vienna BioCenter, Vienna, Austria
[2]Center for Integrative Bioinformatics Vienna (CIBIV), Max Perutz Labs, University of Vienna and Medical University of Vienna, Vienna BioCenter, Vienna, Austria
[3]These authors contributed equally
[4]Current address: Institute of Molecular Biotechnology of the Austrian Academy of Sciences (IMBA), Vienna Biocenter (VBC), 1030 Vienna, Austria
[#]corresponding authors: jason.sims@univie.ac.at, peter.schloegelhofer@univie.ac.at

SUPPLEMENTARY INFORMATION

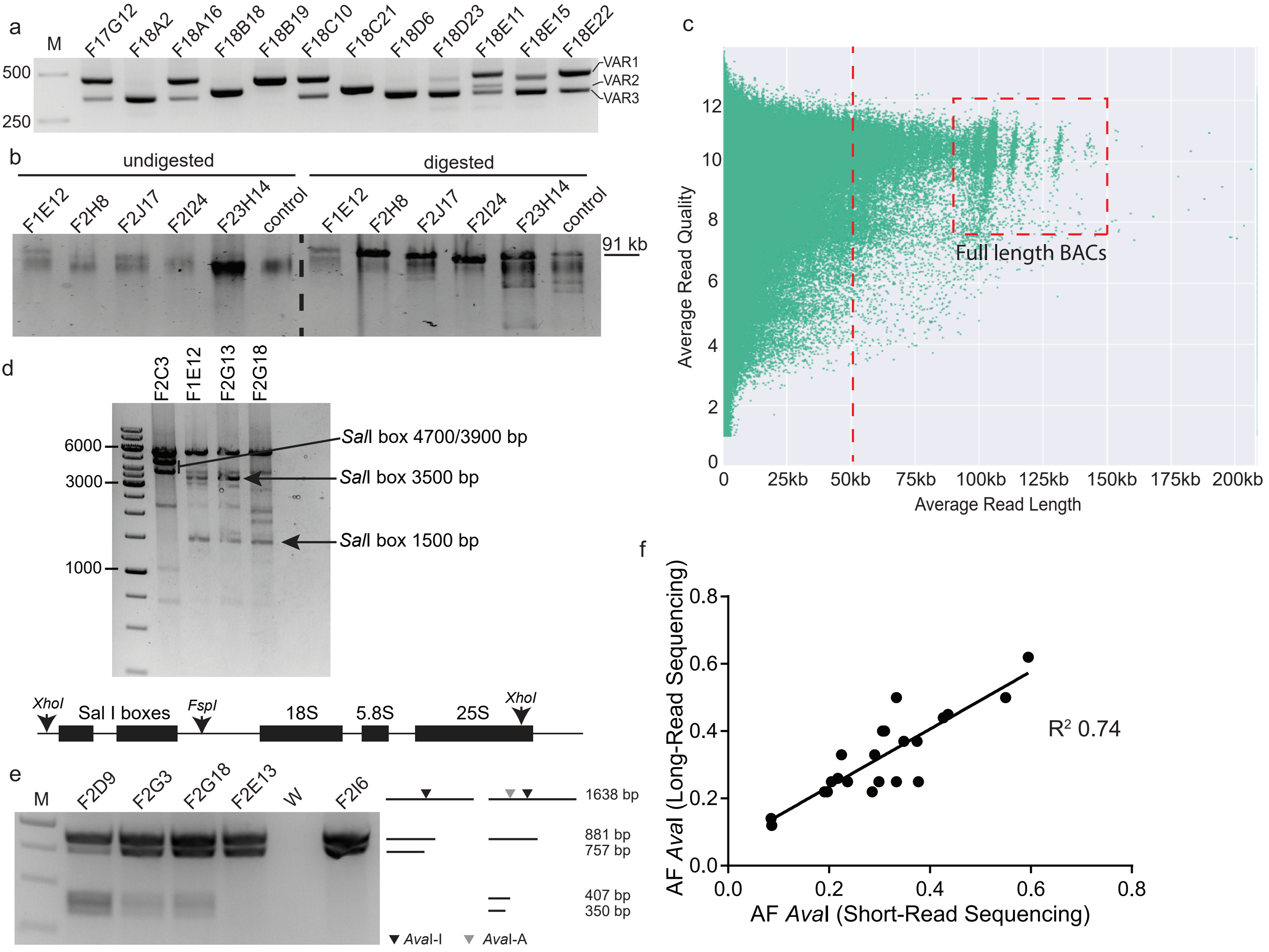

Supplementary Fig. 1: Sequencing and polishing BACs containing rDNA genes

a, Example of an agarose gel depicting the different 3'ETS variants (VAR 1-3) located within BACs. The experiment was repeated twice with similar results. **b**, Example of a pulse field agarose gel with samples of BACs ("control" depicts BAC T15P10 that contains only one rDNA unit) before and after digestion with ApaI which linearizes the BACs without compromising the rDNA units. The BAC F23H14 is of a known size of ~91 kb (GenBank: AC006837.16). The experiment was repeated twice with similar results. **c**, Plot showing an example of a NanoPore multiplex run. The individual reads are plotted according to average read quality vs. read length. Full length sequenced BACs can be visualized as clustered reads of ≥ 90 kb (red dashed box). Red dashed line marks the 50 kb threshold used for the assembly. **d**, Example of a restriction digest gel which confirms the correct assembly of the BACs F2C3, F1E12, F2G13, F2G18. The BACs were digested with XhoI and FspI that cut at the borders of the SalI boxes and within the 25S rDNA. Arrows show the released SalI boxes at the expected sizes. The experiment was repeated twice with similar results. **e**, Example of an agarose gel showing the analysis of the presence/absence of the polymorphism at position 4133 (of the reference rDNA) that creates a CAPS marker that can be cleaved with AvaI. BACs F2D9, F2G3 and F2G18 contain the AvaI site, BACs F2E13 and F2I6 not. Black arrow heads indicate an AvaI site present in all rDNA units (AvaI-I), grey arrow heads indicate the cut site of the allelic AvaI site (AvaI-A). The experiment was repeated twice with similar results. **f**, Correlation analysis of the AvaI allele frequencies derived from either long- or short- read sequencing approaches. Each dot represents an individual assembly.

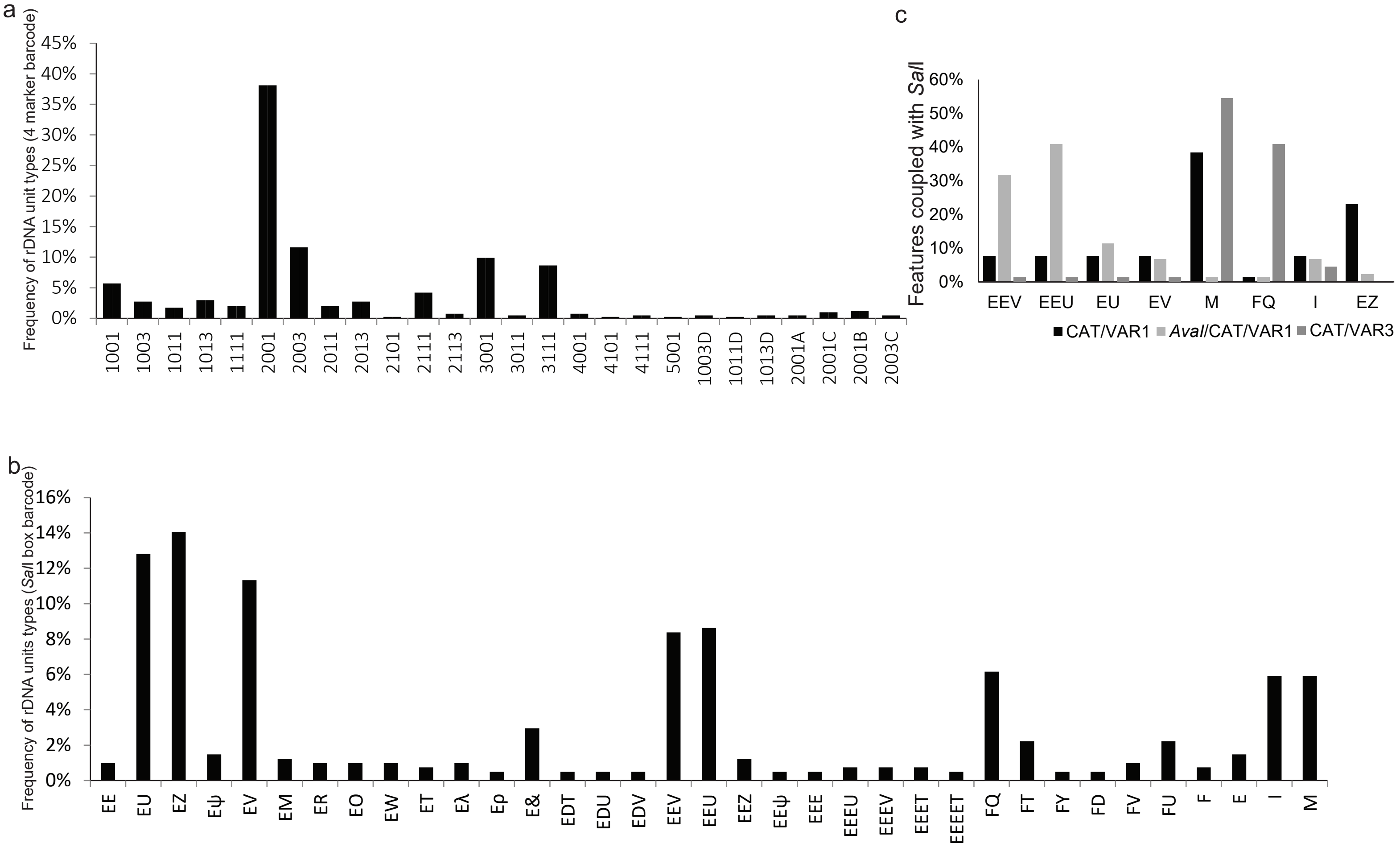

Supplementary Fig. 2: NOR2 contains a heterogeneous population of rDNA units

a, Graph depicting the percentage of rDNA units with the feature combinations: number of promoters (1 to 5), presence (1) or absence (0) of the AvaI restriction site, presence (1) or absence (0) of the CAT polymorphism and length of the 3' ETS variant (1 or 3), presence of the deletion A-D. **b**, Percentage of rDNA variants carrying a certain SalI box combination (see Table 1 for glossary). **c**, Percentage of rDNA variants 011 (black, presence of CAT and VAR1), 111 (dark gray, presence of AvaI, presence of CAT and VAR1) and 013 (light gray, presence of CAT and VAR3) associated with different SalI box combinations (see Table 1 for glossary).

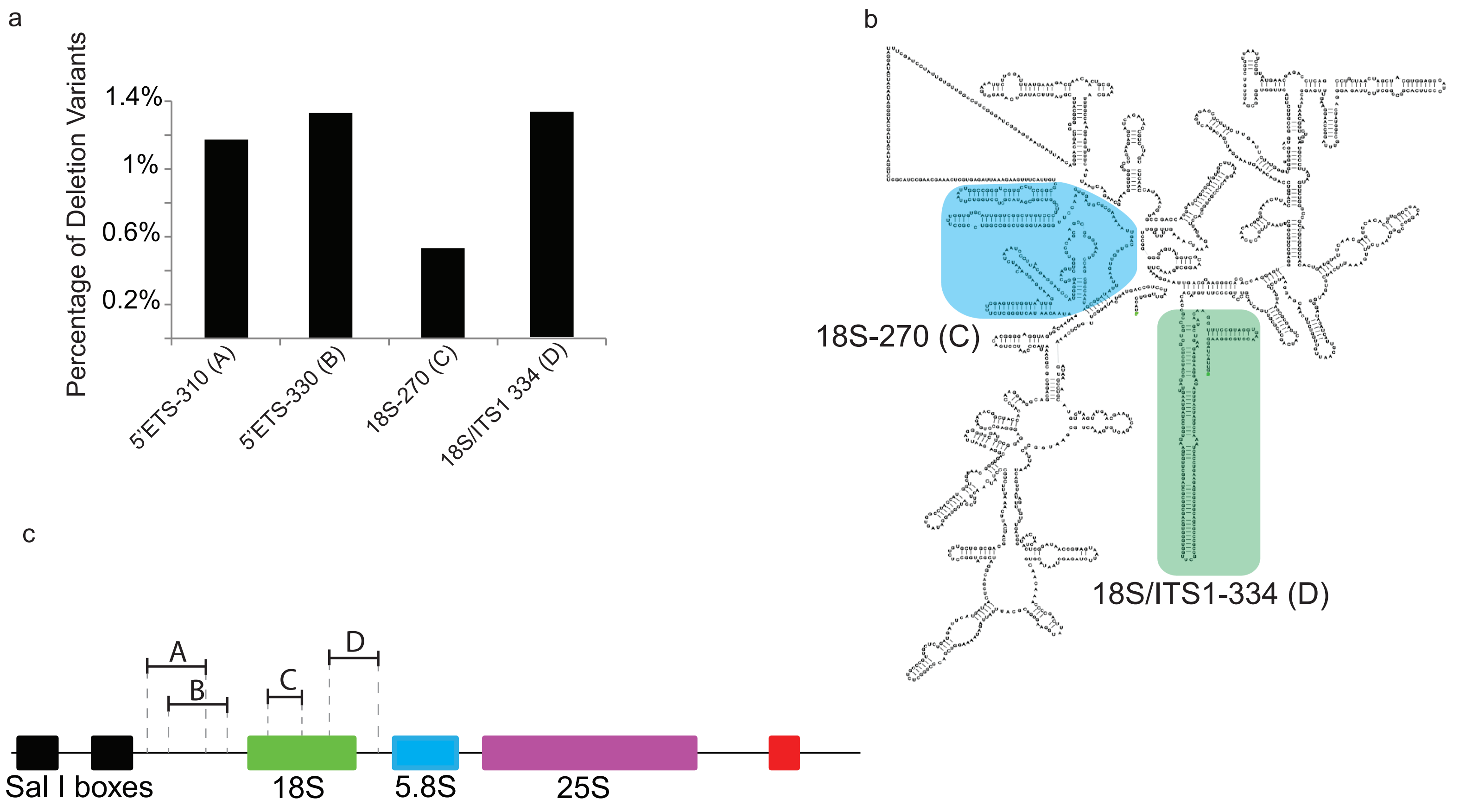

Supplementary Fig. 3: NOR2 contains a heterogeneous population of rDNA units
a, Percentage of rDNA deletion variants within the 5'ETS, 18S and spanning the 18S-ITS1 region. **b**, 2D structure of the 18S rRNA 37. Regions that would be theoretically lost in processed 18S rRNA transcribed from units encoding deletions are highlighted. Light blue shows the 270 bp deletion within the 18S[19] ("C"), light green shows the 334bp deletion that spans the 18S and the ITS1 ("D"). **c**, Graphical depiction of an rDNA unit with identified deletions indicated.

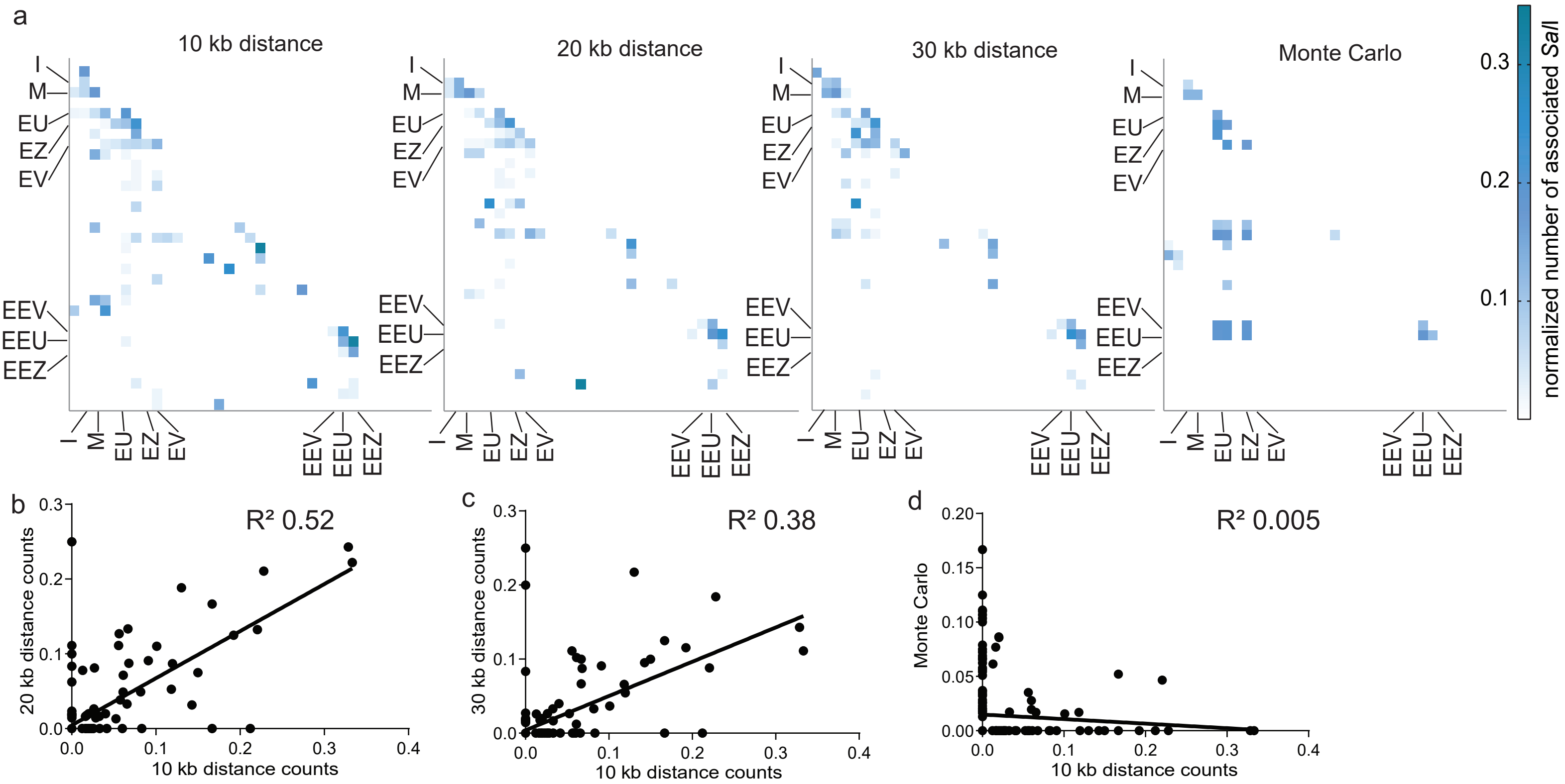

Supplementary Fig. 4: NOR2 is organized in distinct rDNA sub-clusters

a, Heat maps depicting the number of associated rDNA units carrying different SalI box combinations at a 10kb (immediate neighbor), 20kb (neighbor one unit downstream) or 30kb (neighbor two units downstream) distance. The random control (10kb) was performed following a Monte Carlo simulation. Counts were normalized to the numbers of involved rDNA units. Blue color depicts the highest count (0.35) and whites the lowest (0). *SalI* boxes are listed as follows for each heat map on the y-axis from top to bottom and for the x-axis from left to right: EE, EU, EZ, Eψ, EV, EM, ER, EO, EW, ET, Eλ, Eρ, E&, EDT, EDU, EDV, EEV, EEU, EEZ, EEψ, EEE, EEEU, EEEV, EEET, EEEET, FQ, FT, FY, FD, FV, FU, F, E, I, M (see Table 1 for glossary). **b-d**, Scatter plots showing the correlation between the frequencies of associated repeats carrying different SalI box combinations at a 10 kb distance compared to: **b**, 20kb distance (R^2=0.52); **c**, 30 kb distance (R^2=0.38); **d**, Monte Carlo random control (R^2=0.005).

BAC	rDNA variant	Multiplexing – Sample number	Cutoff size (kb)	Reads	Illumina	AF AvaI (Illumina)	AF CAT (Illumina)	AF AvaI (Nanopore)	AF CAT (Nanopore)
F2J17	1	Individually sequenced	> 90	1231	Yes	37.73%	35.02%	25.00%	25.00%
F1E12	1,3	Individually sequenced	> 120	1786	Yes	29.89%	54.82%	25.00%	53.00%
F1A20	1,3	M7886 - (84777)	> 70	135	Yes				
F1C17	1,3	M9626 - (108943)	> 50	182	Yes				
F1C20	1,3	M7869 - (84780)	> 60	65	Yes				
F1A18	3	M7869 - (84781)	> 50	71	Yes				
F2I20	1,3	M7869 - (84785)	> 70	30	Yes	8.50%	6.92%	14.00%	14.00%
F1B23	1	M7869 - (84787)	> 80	80	Yes				
F1D8	1	M8410 - (93327)	> 70	63	Yes	59.50%	56.02%	62.00%	62.00%
F1E1	1	M8410 - (93328)	> 70	40	Yes	33.37%	39.08%	50.00%	50.00%
F1E20	1	M8410 - (93329)	> 60	93	Yes				
F1F4	1,3	M8410 - (93330)	> 60	283	Yes		29.53%		22.00%
F1F5	1,3	M8410 - (93331)	> 70	108	Yes	19.67%	16.18%	22.00%	22.00%
F1F11	1	M8410 - (93332)	> 50	63	Yes	31.00%	39.96%	40.00%	40.00%
F1F16	1	M8410 - (93333)	> 60	158	Yes		7.92%		10.00%
F1F17	1	M8410 - (93334)	> 50	354	Yes	23.65%	21.26%	25.00%	25.00%
F1G11	1,3	M8410 - (93335)	> 60	48	Yes		24.92%		28.00%
F1I9	1,3	M8410 - (93336)	> 40	132	Yes	28.56%	26.89%	22.00%	22.00%
F1K15	1,3	M8410 - (93337)	> 70	103	Yes		36.70%		37.50%
F1H16	1	M8713 - (97105)	> 80	264	Yes				
F1H23	1,3	M8526 - (95136)	> 70	108	Yes				
F1J10	1	M8526 - (95138)	> 70	346	Yes				
F1K10	1	M8526 - (95139)	> 70	405	Yes	55.01%	48.12%	50.00%	50.00%
F1L21	1,3	M8526 - (95141)	All reads	3748	Yes		10.54%		20.00%
F1M1	1	M8526 - (95142)	> 70	385	Yes	37.43%	33.95%	37.00%	37.00%
F1N7	1	M9626 - (108940)	> 50	114	Yes	34.83%	32.39%	37.00%	37.00%
F2I6	1,3	M8713 - (97104)	> 70	626	Yes		28.76%		38.00%
F1P17	1	M8713 – (97107)	> 40	2010	Yes				
F2C3	1	M8713 - (97109)	> 70	228	Yes	43.57%	48.17%	45.00%	45.00%
F2D9	1	M8713 - (97110)	> 80	1334	Yes	42.66%	40.13%	44.00%	44.00%
F2E13	1,3	M8713 - (97111)	> 60	378	Yes				
F2G13	1,3	M8713 - (97113)	> 80	183	Yes				
F2G18	1,3	M8713 - (97114)	> 80	1941	Yes	30.60%	80.15%	40.00%	90.00%
F2G3	1,3	M8713 - (97115)	> 80	325	Yes	8.66%	10.04%	12.00%	20.00%
F2I8	1,3	M9344 - (103502)	> 60	136	Yes	19.08%	20.03%	22.00%	22.00%
F2J3	1	M9344 - (103502)	> 60	112	Yes		9.36%		10.00%
F2J9	1	M9886 - (112593)	> 50	170	Yes				
F2J21	1,3	M9886 - (112592)	> 50	232	Yes	33.37%	67.57%	25.00%	75.00%
F2M18	1,3	M9344 - (103507)	> 60	57	Yes	20.46%	9.85%	25.00%	12.50%
F2L11	1,3	M9886 - (112591)	> 50	206	Yes		21.54%		20.00%
F2L18	1,3	M9344 - (103509)	> 70	149	Yes		18.41%		28.00%
F2L21	1,3	M9344 - (103510)	> 60	83	Yes	21.74%	19.28%	26.00%	26.00%
F2M10	1	M9344 - (103511)	> 60	135	Yes	29.04%	25.78%	33.00%	33.00%
F2M12	1	M9344 - (103512)	> 70	41	Yes		9.18%		11.00%
F2M13	1	M9344 - (103513)	> 60	93	Yes	22.49%	20.39%	33.00%	33.00%
F2N4	1	M9386 - (104608)	> 50	191	Yes				
F2O12	1,3	M9386 - (104609)	> 60	48	Yes		36.28%		28.00%
F2O18	1,3	M9886 - (112589)	> 50	45	Yes	15.73%	16.05%		20.00%
F2O4	1	M9626 - (108947)	> 50	318	Yes		20.92%		25.00%
F2P1	1,3	M9386 - (104613)	> 60	63	No				
F16K18	1	M9886 - (112595)	> 50	126	No				
F16L16	1,3	M10004 - (114460)	> 50	154	No				
F16L21	1,4	M10004 - (114461)	> 50	21	No				
F17A7	1	M10004 - (114464)	> 50	90	No				
F16P19	1	M10004 - (114465)	> 50	469	No				
F19A6	1,3	M10084 - (115248)	> 50	50	No				
F18E22	1,3	M10084 - (115249)	> 50	77	No				
F18E11	1,3	M10084 - (115250)	> 50	58	No				
F18C10	1,3	M10084 - (115251)	> 50	77	No				

Supplementary Table 1: Sequencing and polishing BACs containing rDNA contigs
List of all BACs sequenced with their rDNA variant type, sample number, read cut-off size, number of reads used for the assembly and allelic frequency of two SNP/InDels (*Ava*I and CAT) derived from the Illumina sequencing (Illumina) and the long-read assembly (NanoPore)

		Allele Frequencies							
	POS	WG	AL	YL	INFLO	S	AL-DB	YL-DB	INFLO-DB
25S	4721	0.0051	0.00%	0.00%	0.00%	0.00%	0.00%	0.00%	0.00%
	4866	0.0052	0.00%	0.00%	0.00%	0.00%	0.00%	0.00%	0.00%
	4897	0.0037	0.00%	0.00%	0.00%	0.00%	0.00%	0.00%	0.00%
	4934	0.0147	0.00%	0.00%	0.00%	0.00%	0.00%	0.00%	0.00%
	5157	0.0183	0.00%	0.00%	0.00%	0.00%	0.00%	0.00%	0.00%
	5265	0.0067	0.00%	0.00%	0.00%	0.00%	0.00%	0.00%	0.00%
	5335	0.0062	0.00%	0.00%	0.00%	0.00%	0.00%	0.00%	0.00%
	5356	0.0075	0.00%	0.00%	0.00%	0.00%	0.00%	0.00%	0.00%
	5515	0.0028	0.00%	0.00%	0.00%	0.00%	0.00%	0.00%	0.00%
	5736	0.0219	1.74%	2.17%	1.11%	2.00%	4.23%	0.78%	4.47%
	5932	0.0036	0.00%	0.00%	0.00%	0.00%	0.00%	0.00%	0.00%
	6065	0.0031	0.00%	0.00%	0.00%	0.00%	0.00%	0.00%	0.00%
	6082	0.0091	0.00%	0.00%	0.00%	0.00%	0.00%	0.00%	0.00%
	6255	0.0075	0.00%	0.00%	0.00%	0.00%	0.00%	0.00%	0.00%
	6256	0.0075	0.00%	0.00%	0.00%	0.00%	0.00%	0.00%	0.00%
	6389	0.0054	0.00%	0.00%	0.00%	0.00%	0.00%	0.00%	0.00%
	6413	0.0084	0.00%	0.00%	0.00%	0.00%	0.00%	0.00%	0.00%
	6582	0.0035	0.00%	0.00%	0.00%	0.00%	0.00%	0.00%	0.00%
	6680	0.0345	7.00%	8.13%	8.48%	4.81%	5.57%	3.69%	5.91%
	6712	0.0034	0.00%	0.00%	0.00%	0.00%	0.00%	0.00%	0.00%
	6748	0.0033	0.00%	0.00%	0.00%	0.00%	0.00%	0.00%	0.00%
	6759	0.0067	0.00%	0.00%	0.00%	0.00%	0.51%	0.00%	0.00%
	6888	0.0044	0.00%	0.00%	0.00%	0.00%	0.00%	0.00%	0.00%
	7172	0.0067	0.00%	0.00%	0.00%	0.00%	0.00%	0.00%	0.00%
	7181	0.0051	0.00%	0.00%	0.00%	0.00%	0.00%	0.00%	0.00%
	7382	0.0075	0.00%	0.00%	0.00%	0.00%	1.51%	0.00%	0.00%
	7395	0.0188	0.00%	0.00%	0.00%	0.00%	0.00%	0.00%	0.00%
	7422	0.003	0.00%	0.00%	0.00%	0.00%	0.00%	0.00%	0.00%
	7440	0.0053	1.57%	0.00%	1.62%	0.00%	4.44%	0.00%	3.03%
	7588	0.0063	0.00%	0.00%	0.00%	0.00%	0.00%	0.00%	0.00%
	7847	0.0096	4.72%	5.01%	5.03%	3.28%	0.00%	1.55%	0.00%
	7869	0.0057	1.03%	1.20%	0.00%	0.00%	3.62%	0.70%	0.00%
	7902	0.0084	0.00%	0.00%	3.99%	0.00%	0.00%	0.00%	0.00%
	7987	0.0049	0.00%	0.00%	0.00%	0.00%	0.00%	0.00%	0.00%

Supplementary Table 2: rRNA variants are differentially expressed between tissues
List of the allelic frequencies, based on the ribosome capture RNA-seq and online retrieved polysome sequencing profiles, occurring along the 25S rRNA for each tissue analyzed. Positions (POS), whole genome (WG), adult leaves (AL), young leaves (YL), inflorescences (INFLO), siliques (S), adult leaves online data base (AL-DB), young leaves online data base (YL-DB), inflorescences online data base (INFLO-DB).

Supplementary Table 3: BACs assemblies and accession number
List of all BACs and their accession number as deposited on the NCBI server.

BAC	Accesion Number
f1a18	MT602556
f1a20	MT602557
f1b23	MT602558
f1c17	MT602559
f1c20	MT602560
f1d8	MT602561
f1e1	MT602562
f1e12	MT602563
f1e20	MT602564
f1f4	MT602565
f1f5	MT602566
f1f11	MT602567
f1f16	MT602568
f1f17	MT602569
f1g11	MT602570
f1h16	MT602571
f1h23	MT602572
f1i9	MT602573
f1j10	MT602574
f1k10	MT602575
f1k15	MT602576
f1l21	MT602577
f1m1	MT602578
f1n7	MT602579
f1p17	MT602580
f2c3	MT602581
f2d9	MT602582
f2e13	MT602583
f2g3	MT602584
f2g13	MT602585
f2g18	MT602586
f2i6	MT602587
f2i8	MT602588
f2i20	MT602589
f2j3	MT602590
f2j9	MT602591
f2j17	MT602592
f2j21	MT602593
f2l11	MT602594
f2l18	MT602595
f2l21	MT602596
f2m10	MT602597
f2m12	MT602598
f2m13	MT602599
f2m18	MT602600
f2n4	MT602601
f2o4	MT602602
f2o12	MT602603
f2o18	MT602604
f2p1	MT602605
f16k18	MT602606
f16l16	MT602607
f16l21	MT602608
f16p19	MT602609
f17a7	MT602610
f18c10	MT602611
f18e11	MT602612
f18e22	MT602613
f19a6	MT602614

Supplementary Table 4: Nanopore Runs
List of all Nanopore sequencing runs and their details

Multiplex	Number of Reads	Base Pairs	Quality mean/median	Reads/bp > Q7	Read Length N50 / mean in bp
10084	573 K	2,3 GB	10.1/10.6	473.2K/2GB	9199/3955.21
10004	933.6 K	8.5 GB	11.4/12.1	841.4K/8GB	15667/9121.3
9886	1.1 M	6.9 GB	11.0/11.07	974.6K/6.5GB	16749/6362.15
9626	2.3M	7.4 GB	9.7/9.5	1.9M/6.6M	14093/3272.35
9386	2.9M	10.7GB	10.1/9.8	2.6M/9.9GB	10222/3636.69
9344	6978K	4.5GB	9.8/10.2	613.2K/4.2GB	15450/6400.05
8713	777.8K	6.6GB	9.2/9.8	675.5K/6.2GB	27379/8421.99
8526	2.3M	14.6GB	6.5/6.5	939.8K/6.8GB	13712/6347.05
8410	1.6M	6GB	8.7/9.1	1.3M/5.3GB	9962/3701.86
7886	381.6K	3.7GB	9.2/9.9	324.8K/3.5GB	27213/9745.63
