# Supplementary material for: Sequencing of the Arabidopsis NOR2 reveals its distinct organization and tissue-specific rRNA ribosomal variants
Source: Nat Commun. 2021 Jan 15;12:387. doi: 10.1038/s41467-020-20728-6 (PMC7810690; doi:10.1038/s41467-020-20728-6)
Supplement: Supplementary file 3 — Description of Additional Supplementary Files [file 41467_2020_20728_MOESM3_ESM.pdf]

Description of Additional Supplementary Files

Supplementary Data 1: NOR2 contains a heterogeneous population of rDNA units List of all allelic frequencies per BAC occurring at positions relative to the reference rDNA repeat. Position (POS).

Supplementary Data 2: Unequal distribution of SNP/InDels between NOR2 and the whole genome List of the allelic frequencies, based on Illumina sequencing, occurring in the whole genome sequencing data set (Plant) and on NOR2 (BAC derived NOR2).

Supplementary Data 3: Unequal distribution of SNP/InDels between NOR2 and NOR4 List of all NOR2 called SNP/InDels with their position (POS), relative to the reference rDNA, the reference allelic variant (REF), the alternative variant (VAR), the quality threshold determined by LoFreq (QUAL) and the allelic frequency (AF)

Supplementary Data 4: rRNA variants are differentially expressed between tissues List of the allelic frequencies, based on the total RNA-seq and online retrieved total RNA-seq, occurring along the rDNA reference for each tissue analyzed. Positions (POS), whole genome (WG), adult leaves (AL), young leaves (YL), inflorescences (INFLO), siliques (S), adult leaves online data base (AL-DB), young leaves online data base (YL-DB), inflorescences online data base (INFLO-DB).

Supplementary Data 5: rDNA reference repeat sequence Sequence of the reference rDNA used in this study.

Supplementary Data 6: rDNA units categorized by their features List of all BACs with the units organized according to their position and categorized by the four feature combinations including the *Sal*I repeats.
