# Supplementary material for: Sequencing of the Arabidopsis NOR2 reveals its distinct organization and tissue-specific rRNA ribosomal variants
Source: Nat Commun. 2021 Jan 15;12:387. doi: 10.1038/s41467-020-20728-6 (PMC7810690; doi:10.1038/s41467-020-20728-6)
Supplement: Supplementary file 8 — Supplementary Dataset 5 [file 41467_2020_20728_MOESM8_ESM.docx]

>rDNA_reference

CCAGGACGGCCGGATGTCCGAGAAAAAAAAATGTTGCCGAATAACTTTCGAAAATCATTGGATATGATGCAATGTTTTGTGATCGAATCTCTTAAAATACATCAATAAAGAGTTTAGGATGTCAAGTTTGCATCAAATATGCCCACGGAGCCCCAACTAGACCATGAAAATCCGATGTTGTATCAGGTCAAATGACCTAGCTAGAGGTGTCAAAAAATTATGAAAATTTACCAGAAAATAGGATTTAGTATCCTTATGATGCATGCCAAAAAGAATTTTCAAATTCCAAGTATTTCTTTTTTTTTGGCACCGGTGTCTCCTCAGACATTTCAATGTCTGTTGGTGCCAAGAGGGAAAAGGGCTATTAAGCTATATAGGGGGGTGGGTGTTGAGGGAGTCTGGGCAGTCCGTGGGGAACCCCCTTTTCGGTTCGGACTTGGGTAGCGATCGAGGGATGGTATCGGATATCGGCACGAGGAATGACCGACCGTCCGGCCGCCGGGATTTTCGCCGGAAAACTTTTCCGGCGACTTTTCCGGCGATCGGTTTTGTTGCCTTTTTCCGAGTTTTCTCAGCAGTTCTCGGACAAAAACTGCTGAATCGTCGAGGAGAATGGGCTTGCCTTGCGTGGGCTGCCATTAGTTCTTCGAGGCGTTAGGGTGGCGGCGGTATAAAAGTGTCGGAGTTTTTTCAGCAGTTCTCGGACAAAAATTGCTGAGTGGCCGAGAAGAATGGGCGTGTCATGCGTGGGCTGACATGGATTCTTCGAGGCCTAGGGGTGGCGGTATATAACTTGTTCGCATGATATTACCGAGATGTCCCCACGGGCATCTTTTCACCTCGTCGCCGAAGAGAATGGGCGTGTCATGGCATGGGCTGACATGGATTCTCCTAGGCCGTTTGGGTGGCGGTATAGTCGTCTTGCGCACGAAATACCGAGATGTCCCCATGGGCATCGATTCCACCCGCCTAGGTTGGATGGGCGTGCTTCGTCGGAAAGCATGGATCCGCCTAGGCTGTCCCGAGTGTGAGCGAGGTGTGAGTGTCGCCCATGGGCATCGACACCTTGCGGCTAGGAACTGGAACGAGACGGGTGGCAAAGATTTCGAGTAGCACTTCATACTACCGTGGGTTTTTTAAACCTTCCGAGTTTTGTTGATGTTATTCCGAGAATTAGCAAACCGTAACGAAGATGTTCTTGGCAACCATCTTTTGATGGGAGTCCGGCTGTTCGATAGCCGGCCAAGGGTGATGAACGAAATGTGAACCCTTGTCTCGCCTAGGTTGGATGGGCGTGCTTCGTTGGAAAGCATGGATCCGCCTAGGCTGTCCCGAGTGTGAGCGAGGTGTGAGTGTCGCCCATGGGCATCGACACCTTGCGGCTAGGAACTGGAACGAGACGGGTAGCAAAGATTTCGAGTAGCACTTCATACTACCGTGGGTTTTTTAAACCTTCCTAGTTTTGTTGATGTTATTCCGAGAATTAGCAAACCGTAACGAAGATGTTCTTGGCAACCATCTTTTGATGGGAGTCCGGCTGTTCGAAAGCCGGCCAAGGGTGATGAACGAAATGTGAACCCTTGTCTCGCCTAGGTTGGATGGGCGTGCTTCGTTGGAAAGCATGGATCCGCCTAGGCTGTCCCGAAGGTATCTCGCGCTTGTACGGCTTTGGCTCGGATTCGTCCGTCTTCTTTCTTCTTAGCCGAGTACTTCGGTAGATTAGTTGGAACGATTGATGATTTTGAGTTAATTGAACGTTCGGCGTATGAGTGGTGATCGGATAGCTAGTGTTCGTAGGCTCCATGCTCGCGCATCGAACTACCTACCACCTATCCTTCTCAGTTAATTCACGGGCGATGTTACGCTCGATGATGAGTTCCGGGGCCTGTGTTTCGTACCTAATTTGAAGGAATTGTTGAGTTTGGTTTACACCTTTGCCCGCGGCTTCTCCTTCGTGGGGAAGTCGTGGGCACAAACATCGGCGCTTGTTCACCTCTCGTCATCGCATTTGTTGCCTTGCTCGCATTGGTGAATGAGTTGCGGGTTGAAATCTCGGATGCGGAAAAGTTGTCGACGGTGACTCGAAGTGATTCAGTCCCGCCAAAGCTCATCCGTCCTTCGGGCAAAAGATGACGGTCAAGACCTCGTCCTTTCTCTCTTTCCATTGCGTTTGAGAGGATGTGGCGGGGAATTGCCGTGATCGATGAATGCTACCTGGTTGATCCTGCCAGTAGTCATATGCTTGTCTCAAAGATTAAGCCATGCATGTGTAAGTATGAACGAATTCAGACTGTGAAACTGCGAATGGCTCATTAAATCAGTTATAGTTTGTTTGATGGTAACTACTACTCGGATAACCGTAGTAATTCTAGAGCTAATACGTGCAACAAACCCCGACTTATGGAAGGGACGCATTTATTAGATAAAAGGTCGACGCGGGCTCTGCCCGTTGCTCTGATGATTCATGATAACTCGACGGATCGCATGGCCTCTGTGCTGGCGACGCATCATTCAAATTTCTGCCCTATCAACTTTCGATGGTAGGATAGTGGCCTACCATGGTGGTAACGGGTGACGGAGAATTAGGGTTCGATTCCGGAGAGGGAGCCTGAGAAACGGCTACCACATCCAAGGAAGGCAGCAGGCGCGCAAATTACCCAATCCTGACACGGGGAGGTAGTGACAATAAATAACAATACCGGGCTCTTTCGAGTCTGGTAATTGGAATGAGTACAATCTAAATCCCTTAACGAGGATCCATTGGAGGGCAAGTCTGGTGCCAGCAGCCGCGGTAATTCCAGCTCCAATAGCGTATATTTAAGTTGTTGCAGTTAAAAAGCTCGTAGTTGAACCTTGGGATGGGTCGGCCGGTCCGCCTTTGGTGTGCATTGGTCGGCTTGTCCCTTCGGTCGGCGATACGCTCCTGGTCTTAATTGGCCGGGTCGTGCCTCCGGCGCTGTTACTTTGAAGAAATTAGAGTGCTCAAAGCAAGCCTACGCTCTGGATACATTAGCATGGGATAACATCATAGGATTTCGATCCTATTGTGTTGGCCTTCGGGATCGGAGTAATGATTAACAGGGACAGTCGGGGGCATTCGTATTTCATAGTCAGAGGTGAAATTCTTGGATTTATGAAAGACGAACAACTGCGAAAGCATTTGCCAAGGATGTTTTCATTAATCAAGAACGAAAGTTGGGGGCTCGAAGACGATCAGATACCGTCCTAGTCTCAACCATAAACGATGCCGACCAGGGATCAGCGGATGTTGCTTATAGGACTCCGCTGGCACCTTATGAGAAATCAAAGTTTTTGGGTTCCGGGGGGAGTATGGTCGCAAGGCTGAAACTTAAAGGAATTGACGGAAGGGCACCACCAGGAGTGGAGCCTGCGGCTTAATTTGACTCAACACGGGGAAACTTACCAGGTCCAGACATAGTAAGGATTGACAGACTGAGAGCTCTTTCTTGATTCTATGGGTGGTGGTGCATGGCCGTTCTTAGTTGGTGGAGCGATTTGTCTGGTTAATTCCGTTAACGAACGAGACCTCAGCCTGCTAACTAGCTACGTGGAGGCATCCCTTCACGGCCGGCTTCTTAGAGGGACTATGGCCGTTTAGGCCAAGGAAGTTTGAGGCAATAACAGGTCTGTGATGCCCTTAGATGTTCTGGGCCGCACGCGCGCTACACTGATGTATTCAACGAGTTCACACCTTGGCCGACAGGCCCGGGTAATCTTTGAAATTTCATCGTGATGGGGATAGATCATTGCAATTGTTGGTCTTCAACGAGGAATTCCTAGTAAGCGCGAGTCATCAGCTCGCGTTGACTACGTCCCTGCCCTTTGTACACACCGCCCGTCGCTCCTACCGATTGAATGATCCGGTGAAGTGTTCGGATCGCGGCGACGTGGGTGGTTCGCCGCCCGCGACGTCGCGAGAAGTCCACTAAACCTTATCATTTAGAGGAAGGAGAAGTCGTAACAAGGTTTCCGTAGGTGAACCTGCGGAAGGATCATTGTCGATACCTGTCCAAAACAGAACGACCCGCGAACCAAAGATCACCACTCTCGGTGGGCCGGTTTCTTAGCCGATTCCTTGCCCGCCGGATCCGTGGTTTCGCGTATCGGCATGATCGGGAGCTTTTATCTCGGTCTTGTCGTGCGCGTTGCTTCCGGATATCACAAAACCCCGGCACGAAAAGTGTCAAGGAACATGCAAACGAACGGCTGGCATTCGCCTCCCCGGAGACGGAGTGTGGGCGGATGCTGTGCTGCGAACTGAAGTCTAAAACGACTCTCGGCAACGGATATCTCGGCTCTCGCATCGATGAAGAACGTAGCGAAATGCGATACTTGGTGTGAATTGCAGAATCCCGTGAACCATCGAGTCTTTGAACGCAAGTTGCGCCCCAAGCCTTCTGGCCGAGGGCACGTCTGCCTGGGTGTCACAAATCGTCGTCCCTCACCATCCTTTGCTGATGCGGGACGGAAGCTGGTCTCCCGTGTGTTACCGCACGCGGTTGGCCTAAATCCGAGCCAAGGACGCCTGGAGCGTACCGACATGCGGTGGTGAACTTGATCCATTACATTTTATCGGTCGCTCTTGTCCGGAAGCTGTAGATGACCCAAAGTCCATATAGCGACCCCAGGTCAGGCGGGATTACCCGCTGAGTTTAAGCATATCAATAAGCGGAGGAAAAGAAACTAACAAGGATTCCCTTAGTAACGGCGAGCGAACCGGGAAGAGCCCAGCTTGAAAATCGGACGTCTTCGGCGTTCGAATTGTAGTCTGGAGAAGCGTCCTCAGCGACGGACCGGGCCTAAGTTCCCTGGAAAGGGGCGCCAGAGAGGGTGAGAGCCCCGTCGTGCCCGGACCCTGTCGCACCACGAGGCGCTGTCTACGAGTCGGGTTGTTTGGGAATGCAGCCCCAATCGGGCGGTAAATTCCGTCCAAGGCTAAATACGGGCGAGAGACCGATAGCGAACAAGTACCGCGAGGTAAAGATGAAAAGGACTTTGAAAAGAGAGTCAAAGAGTGCTTGAAATTGTCGGGAGGGAAGCGGATGGGGGCCGGCGATGCGTCCTGGTCGGATGCGGAACGGAGCAATCCGGTCCGCCGATCGATTCGGGGCGTGGACCGACGCGGATTACGGTGGCGGCCTAAGCCCGGGCTTTTGATACGCTTGTGGAGACGTCGCTGCCGTGATCGTGGTCTGCAGCACGCGCCTAACGGCGTGCCTCGGCATCAGCGTGCTCCGGGCGTCGGCCTGTGGGCTCCCCATTCGACCCGTCTTGAAACACGGACCAAGGAGTCTGACATGTGTGCGAGTCAACGGGTGAGTAAACCCGTAAGGCGCAAGGAAGCTGATTGGCGGGATCCCTCGCGGGTGCACCGCCGACCGACCTTGATCTTCTGAGAAGGGTTCGAGTGTGAGCATGCCTGTCGGGACCCGAAAGATGGTGAACTATGCCTGAGCGGGGTAAAGCCAGAGGAAACTCTGGTGGAAGCCCGCAGCGATACTGACGTGCAAATCGTTCGTCTGACTTGGGTATAGGGGCGAAAGACTAATCGAACCATCTAGTAGCTGGTTCCCTCCGAAGTTTCCCTCAGGATAGCTGGAGCTCGGACGCGAGTTCTATCGGGTAAAGCCAATGATTAGAGGCATTGGGGGCGCAACGCCCTCGACCTATTCTCAAACTTTAAATAGGTAGGACGTGTCGGCTGCTTTGTTGAGCCGTCACACGGAATCGAGAGCTCCAAGTGGGCCATTTTTGGTAAGCAGAACTGGCGATGCGGGATGAACCGGAAGCCGGGTTACGGTGCCCAACTGCGCGCTAACCTAGAACCCACAAAGGGTGTTGGTCGATTAAGACAGCAGGACGGTGGTCATGGAAGTCGAAATCCGCTAAGGAGTGTGTAACAACTCACCTGCCGAATCAACTAGCCCCGAAAATGGATGGCGCTTAAGCGCGCGACCTATACCCGGCCGTCGGGGCAAGAGCCAGGCCTCGATGAGTAGGAGGGCGCGGCGGTCGCTGCAAAACCTAGGGCGCGAGCCCGGGCGGAGCGGCCGTCGGTGCAGATCTTGGTGGTAGTAGCAAATATTCAAATGAGAACTTTGAAGGCCGAAGAGGGGAAAGGTTCCATGTGAACGGCACTTGCACATGGGTTAGTCGATCCTAAGAGTCGGGGGAAACCCGTCTGATAGCGCTTAAGCGCGAACTTCGAAAGGGGATCCGGTTAAAATTCCGGAACCGGGACGTGGCGGTTGACGGCAACGTTAGGGAGTCCGGAGACGTCGGCGGGGGCCTCGGGAAGAGTTATCTTTTCTGTTTAACAGCCTGCCCACCCTGGAAACGGCTCAGCCGGAGGTAGGGTCCAGCGGCTGGAAGAGCACCGCACGTCGCGTGGTGTCCGGTGCGCCCCCGGCGGCCCTTGAAAATCCGGAGGACCGAGTGCCGCTCACGCCCGGTCGTACTCATAACCGCATCAGGTCTCCAAGGTGAACAGCCTCTGGTCGATGGAACAATGTAGGCAAGGGAAGTCGGCAAAATGGATCCGTAACTTCGGGAAAAGGATTGGCTCTGAGGGCTGGGCTCGGGGGTCCCAGTTCCGAACCCGTCGGCTGTCAGCGGACTGCTCGAGCTGCTTCCGCGGCGAGAGCGGGTCGCCGCGTGCCGGCCGGGGGACGGACTGGGAACGGCTCTCTCGGGAGCTTTCCCCGGGCGTCGAACAGTCAGCTCAGAACTGGTACGGACAAGGGGAATCCGACTGTTTAATTAAAACAAAGCATTGCGATGGTCCCTGCGGATGCTAACGCAATGTGATTTCTGCCCAGTGCTCTGAATGTCAAAGTGAAGAAATTCAACCAAGCGCGGGTAAACGGCGGGAGTAACTATGACTCTCTTAAGGTAGCCAAATGCCTCGTCATCTAATTAGTGACGCGCATGAATGGATTAACGAGATTCCCACTGTCCCTGTCTACTATCCAGCGAAACCACAGCCAAGGGAACGGGCTTGGCAGAATCAGCGGGGAAAGAAGACCCTGTTGAGCTTGACTCTAGTCCGACTTTGTGAAATGACTTGAGAGGTGTAGGATAAGTGGGAGCTTCGGCGCAAGTGAAATACCACTACTTTTAACGTTATTTTACTTACTCCGTGAATCGGAGGCGGGGTACAACCCCTGTTTTTGGTCCCAAGGCTCGCTTCGGCGGGTCGATCCGGGCGGAGGACATTGTCAGGTGGGGAGTTTGGCTGGGGCGGCACATCTGTTAAAAGATAACGCAGGTGTCCTAAGATGAGCTCAACGAGAACAGAAATCTCGTGTGGAACAAAAGGGTAAAAGCTCGTTTGATTCTGATTTTCAGTACGAATACGAACCGTGAAAGCGTGGCCTATCGATCCTTTAGACCTTCGGAATTTGAAGCTAGAGGTGtCAGAAAAGTTACCACAGGGATAACTGGCTTGTGGCAGCCAAGCGTTCATAGCGACGTTGCTTTTTGATCCTTCGATGTCGGCTCTTCCTATCATTGTGAAGCAGAATTCACCAAGTGTTGGATTGTTCACCCACCAATAGGGAACGTGAGCTGGGTTTAGACCGTCGTGAGACAGGTTAGTTTTACCCTACTGATGCCCGCGTCGCGATAGTAATTCAACCTAGTACGAGAGGAACCGTTGATTCGCACAATTGGTCATCGCGCTTGGTTGAAAAGCCAGTGGCGCGAAGCTACCGTGCGCTGGATTATGACTGAACGCCTCTAAGTCAGAATCCGGGCTAGAAGCGACGCATGCGCCCGCCGCCCGATTGCCGACCCTCAGTAGGAGCTTAGGCTCCCAAAGGCACGTGTCGTTGGCTAAGTCCGTTCGGCGGAAGCGTCGTTCGGACCGCCTTGAATTATAATTACCACCGAGCGGCGGGTAGAATCCTTTGCAGACGACTTAAATACGCGACGGGGTATTGTAAGTGGCAGAGTGGCCTTGCTGCCACGATCCACTGAGATTCAGCCCTTTGTCGCTAAGATTCGACCCTCCCCTAAATCACTCCAAAAAAAACAATCCCCAATTCTACACAAGTGTTTCTACACTAACAAAGCAACAGCTCCTTAACGAATTCCCAACTTTACACGAGCTCGTCTCTCGAGGTTAAATGTTATTACTTGGTAAGATTCCGGACCTCGCCAAGTGTTTTGAAAACCCGCAACGCTCGCAAAGGTGGATAGTGAGAATAATAAGTGAAGAGACAGACTTGTCCAAAACGCCCACCACGAAGGTGCATAGTGAGAAGAGTAAGTCAAGAGATAGACTTGTCCAAAAAGAAACGGAAGAGAAAGCGTGGGGAGACGCTCACGAAGGTGCATAGTGAGAAGAGTAAGTCAAGAGACAGACTTGTTCGAAAAGAAACAGAAGAGAATGCTTGGGGTTACACTCACGAAGGTGCATAGTGAGAAGAGTAAGTCAAGAGACAGACTTGTTCGAAAAGAAACAAAAGAGAATGCTTGGGGAGATAGAAGTGTGAGATAGTTCTCAAGCTAAGAAAGTTGTAAAAGCTAAGAACTAGCATCAAATGATGGATGAAACACAAGGTAGTTGTTGAAAAGTCAAACACTTGGTGATATGAACACAAACGTTCAATATGACAAACCCATGCCAAGTAAAGAGAAAATGAAAACTGGTGATTGTTGCGGAAATCGTCCAGGATTCCTCGACCAGGACTTGAAATCGTCGAGGGGAAAAAATCGGTTCCGAGGAATCGTCGATCCGGACTTGGAATCGTCGAGAAAAGTTTACCGGGTCCGAGGATTT
