# Supplementary material for: Sequencing of the Arabidopsis NOR2 reveals its distinct organization and tissue-specific rRNA ribosomal variants
Source: Nat Commun. 2021 Jan 15;12:387. doi: 10.1038/s41467-020-20728-6 (PMC7810690; doi:10.1038/s41467-020-20728-6)
Supplement: Supplementary file 9 — Supplementary Dataset 6 [file 41467_2020_20728_MOESM9_ESM.pdf]

Supplementary File 3: rDNA units categorized by their features

Barcoded Contigs

F2D9

	1	2	3	4	5	6	7	8	9
Position	0 - 17650	17650 - 28737	28738 - 39827	39828 - 50909	50910 - 61972	61973 - 73031	73032 - 84686	84687 - 95738	95739 - 101788
	3(EEV)111	3(EEV)001	3(EEV)111	3(EEV)001	3(EEU)011	3(EEU)111	4(EEEU)101	3(EDU)001	Incomplete

F1P17

	1	2	3	4	5	6	7	8
Position	77262 - 86955	66575 - 77261	56188 - 66574	45257 - 56187	34568 - 45256	23653 - 34567	12975 - 23652	3280 - 12974
	2(EE)001	2(EZ)001	2(EZ)001A	2(Eλ)001	2(EZ)001	2(EΨ)001	2(EY)001	2(EE)001

F2C3

	1	2	3	4	5	6	7	8	9	10	11
Position	124792 - 135868	113713 - 124791	102023 - 113712	90945 - 102022	79259 - 90944	68794 - 79868	57720 - 68793	46667 - 57719	35605 - 46666	24531 - 35604	13455 - 24530
	3(EEV)001	3(EEV)111	4(EEEV)001	3(EEV)111	3(EEV)011	3(EEV)001	3(EEU)111	3(EEU)001	3(EEU)111	3(EEU)111	3(EEU)001

F2E13

	1	2	3	4	5	6	7	8
Position	98217 - 108412	87641 - 98216	77381 - 87640	66910 - 77380	56441 - 66909	45992 - 56440	35624 - 45991	15937 - 35623
	2(EV)001C	2(EZ)003	2(FQ)001	2(EV)001	2(EV)001	2(EU)001	2(EZ)001	2(EU)001

Note to repeat 8: this repeat is 20000 bp long! It seems that 2 repeats are fused together

F2G3

	1	2	3	4	5	6	7	8
Position	80994 - 91466	70596 - 80993	60247 - 70595	49571 - 60246	40187 - 49570	29728 - 40186	19267 - 29727	8804 - 19266
	2(EV)001	2(EW)003	2(EV)113	2(EZ)001	1(M)011	2(EU)001	2(EU)001	1(EU)001

F2G13

	1	2	3	4	5	6	7	8
Position	8581 - 19260	19260 - 40608	40609 - 51287	51288 - 61965	61966 - 72628	72629 - 83282	83283 - 93947	93948 - 99602
	2(EZ)001	2(EZ)001	2(EZ)001	2(EZ)001	2(EZ)001	2(EZ)001	2(EZ)001	2(EZ)

Note to repeat 8: only the promoter is present

F2G18

	1	2	3	4	5	6	7	8	9	10
Position		21619 - 31752	31753 - 41076	41077 - 50300	50301 - 59566	59567 - 68669	68670 - 77771	77772 - 87037	87038 - 96308	96309 - 105570
	1(M)013	2(FQ)013	1(F)011	1(M)013	1(I)111	1(E)111	1(E)111	1(M)013	1(I)111	1(M)013

F1A18

	1	2	3	4	5	6
Position	8447 - 18769	18769 - 29088	29089 - 39402	39403 - 49699	49700 - 60002	60003 - 70303
	2(FU)003	2(FU)003	2(FT)003	2(FT)003	2(FT)003	2(FT)003
			C	C	T	C

F2I6

	1	2	3	4	5	6	7	8	9	10	11	12	13
Position	7337 - 16791	16789 - 26058	26059 - 36119	36120 - 46270	46271 - 55407	55408 - 65459	65460 - 74503	74504 - 84968	84969 - 93887	93888 - 103155	103156 - 113205	113206 - 123646	123647 - 132866
	1(??)013	1(I)001	2(EM)001	2(FQ)013	1(I)013	2(EM)011	1(M)011D	2(EV)001	1(M)013D	1(I)001	2(EM)001	2(EU)001	1(M)013

Note to repeat 1: the initial part of the repeat is missing

F1A20

	1	2	3	4	5	6
Position	10318 - 20544	20545 - 30768	30769 - 40981	40982 - 51192	51193 - 61749	61750 -
	2(FT)003	2(FT)003	2(FT)003	2(FT)003	2(FY)001	2(ET)003
	C	C	T	C		

F1B23

	1	2	3	4	5	6	7	8	9	10
Position	8583 - 19276	19276 - 29960	29961 - 40650	40651 - 51329	51330 - 62011	62012 - 72666	72667 - 83329	83330 - 93771	93772 - 104440	104441 - 115108
	2(EZ)001	2(EZ)001	2(EZ)001	2(EZ)001	2(EZ)001	2(EZ)001	2(EZ)001	2(EU)001	2(EZ)001	2(EZ)001

F1E1

	1	2	3	4	5	6	7	8
Position	10526 - 21628	21629 - 31959	31960 - 43040	43041 - 54098	54099 - 65154	65155 - 76213	76214 - 87278	87279 - 98342
	3(FEV)111	2(FS)001	3(EEV)111	3(EEV)111	3(EEU)001	3(EEU)111	3(EEU)111	3(EEU)001

F1C20

	1	2	3	4	5	6
Position	13032 - 23348	23349 - 33670	33671 - 43967	43968 - 54267	54268 - 64221	64222 - 74399
	2(FU)003	2(FU)003	2(FU)003	2(EU)003	1(Z)003	2(EO)003

F1E12

	1	2	3	4	5	6	7	8	9	10	11	12
	12165 - 22515	22516 - 33215	33216 - 43083	43084 - 53428	53429 - 64124	64126 - 75041	75042 - 85732	85733 - 96424	96425 - 107115	107116 - 118801	118802 - 129481	129482 - 138284
	2(EV)003	2(EV)001	2(EZ)003C	2(FQ)003	2(EU)111	2(EZ)011	2(EΨ)011	2(EZ)111	2(EZ)011	2(EZ)011	4(EEEU)111	2(EZ)001

F1F4

	1	2	3	4	5	6	7	8	9
	10574 - 19834	19835 - 30303	30304 - 39230	39231 - 49366	49307 - 58628	58629 - 68665	68666 - 78788	78789 - 88037	88038 - 98478
	1(I)001	2(EU)001	1(M)003D	2(FQ)013	1(I)001	2(EM)001	2(FQ)013	1(I)001	2(EU)001
								Note to repeat 8: SalI box (I) is 454 bp long	

F1F5

	1	2	3	4	5	6	7	8	9
	95031 - 84541	74390 - 84540	64257 - 74389	53790 - 64256	43375 - 53789	33998 - 43374	23882 - 33997	13434 - 23881	2992 - 13433
	2(EZ)003	2(FQ)003	2(FQ)003	2(EV)111	2(EU)001	1(N)003	1($)003	2(EU)111	2(EU)001
				Note to repeat 4: SalI box(V) is 1054 bp long					

F1F11

	1	2	3	4	5
	16818 - 27895	27895 - 38963	38694 - 50040	50041 - 61079	61080 - 69043
	3(EEV)001	3(EEU)111	3(EEV)001	3(EEU)?11	4(EEEU)00?
	Note to repeat 1: [illegible]	Note to repeat 2: S[illegible]	Note to repeat 3: [illegible]	Note to repeat 4:	Note to repeat 5: it is not possible to determine variant type

F1E20

	1	2	3	4	5	6	7	8
	97540 - 86608	75033 - 86608	64575 - 75032	54420 - 64574	44300 - 54419	34171 - 44299	23823 - 34170	13595 - 23824
	2(Eλ)001	4(EEET)001	2(EV)001	2(EV)001	2(EU)001	2(EU)001	2(EZ)001	2(EW)001

		Note to repeat 2: S	Note to repeat 3: Sall box is 1050 bp long				Note to repeat 7: Sall box (Z) is 1250 bp long			
F1F16										
	1	2	3	4	5	6	7	8		
	1(M)001	2(EU)001	2(EU)001	2(EY)001	1(M)011	2(EU)001	2(EU)001	2(EU)001		
F1G11										
	1	2	3	4	5	6	7	8		
	72154 - 62094	52045 - 62094	41725 - 52044	32481 - 41724	23218 - 32480	13190 - 23217	7316 - 13189			
	2(E&)011	2(E&)001	2(ES)001	1(M)013	1(I)001	2(E&)001	2(EΨ)0??			
	Note to repeat 1: Sall box (E) is 299 bp long			Note to repeat 4: Sall box (M) is 553 bp long						
F1H16										
	1	2	3	4	5	6	7	8	9	10
	12329 - 22493	22494 - 33481	33482 - 44155	44156 - 53852	53853 - 64521	64522 - 74900	74901 - 85808	85809 - 96478	96479 - 107371	107372 - 118033
	2(EV)001B	2(ELV)001B	2(EV)001	2(EE)001	2(EV)001	2(EV)001B	2(EΨ)001	2(EV)001	2(EV)001	2(EZ)001
	Note to repeat 1: S	Note to repeat 2: Sall box V is 1050 bp long								Note to repeat 10: Sall box (Z) is 1252 bp long
F1I9										
	1	2	3	4	5	6	7	8	9	
	16260 - 27260	27260 - 36717	36718 - 47118	47119 - 57788	57789 - 68333	68334 - 78478	78479 - 88587	88588 - 98170	98171 - 108747	
	2(Eρ)003	2(FD)003	2(EU)111	2(EZ)001	2(EZ)113	2(ER)003	2(FQ)003	2(FC)001	2(Eρ)00?	
		Note to repeat 2: Sall box F is 301 bp long								
F1K10										
	1	2	3	4	5	6	7	8	9	
	101665 - 90606	79545 - 90605	68524 - 79554	57489 - 68523	46459 - 57488	34952 - 46458	23989 - 34951	14054 - 23988	3946 - 14053	
	3(EEV)001	3(EEV)111	3(EEV)001	3(EEV)001	3(EEV)111	3(EDT)111	2(EU)001	2(DU)111	2(??)???	
	Note to repeat 1: S	Note to repeat 2: S	Note to repeat 3:	Note to repeat 4:	Note to repeat 5	Note to repeat 6: Sall box rearrangment is wierd. Sall box (D) is 224 bp long				
F1K15										
	1	2	3	4	5	6	7	8		
	10373 - 19629	19629 - 30574	30575 - 39609	39610 - 48871	48872 - 58781	58782 - 68921	68922 - 79079	79080 - 89789		
	1(I)001	2(EΨ)001	1(H)013	1(I)001	2(E&)013	1(Ψ)013	2(DS)001	3(EEΨ)		
F1L21										
	1	2	3	4	5					
	5930 - 15027	15028 - 25450	25450 - 36028	36029 - 45244	45244 - 55473					
	1(F)001	2(EU)001	2(EY)001	1(E)013	2(EU)001					
		Note to repeat 2 Sall box (U) is 1047 b		Note to repeat 4 Sall box (E) is 253 bp long						
F1M1										
	1	2	3	4	5	6	7	8		
	10685 - 21764	21764 - 32827	32828 - 43888	43889 - 54964	54947 - 65977	65978 - 77009	77010 - 88133	88134 - 98853		
	3(EEV)001	3(EEV)111	3(EEV)001	3(EEU)111	3(EEU)001	3(EEU)111	3(EEZ)001	3(EEU)101		
	Note to repeat 1 Sall box (2E) is 298 bp long			Note to repeat 4 Sall box (U) is 1047 bp long			Note to repeat 7 Sall box (U) is 1255 bp long			
F2I20										
	1	2	3	4	5	6	7			
	10382 - 20854	20855 - 33006	33007 - 43171	43172 - 53640	53641 - 64116	64117 - 74369	74370 - 85433			

2(EV)111	5(EEEET)001	2(EV)001B	2(EV)001	2(EV)001	2(EW)001B	2(EU)001

F2J17

1	2	3	4	5	6	7	8
81054 - 91358	69758 - 81053	58687 - 69757	47500 - 58686	36325 - 47399	25253 - 36324	14177 - 25252	3105 - 14176
3(EEE)001	3(EEZ)001	3(EEU)001	3(EEZ)001	3(EEU)111	3(EEU)001	3(EEU)001	3(EEU)111

F1D8

1	2	3	4	5	6	7	8
11075 - 22184	22184 - 33188	33189 - 44865	44866 - 55941	55942 - 66990	66991 - 78016	78017 - 88446	88447 - 99508
3(EEV)001	3(EDV)111	4(EEEV)111	3(EEV)001	3(EEU)111	3(EEU)111	2(EU)001	3(EEU)111
Noteto repeat 1. S	Note to repeat 2. Sall box (E) is 299 bp long						

F1F17

1	2	3	4	5	6	7	8
12341 - 23424	23425 - 34505	34506 - 45799	45800 - 56909	56910 - 68137	68138 - 79239	79240 - 90242	90243 - 101300
3(EEV)111	3(EEV)001	3(EEZ)001	3(EEU)001	3(EEU)001	3(EEU)111	3(EEU)001	3(EEU)001
Note to repeat 1: !	Note to repeat 2: Sall box (V) is 1056 bp long						

F1H23

1	2	3	4	5	6	7	8
82266 - 92167	71893 - 82265	61206 - 71892	51063 - 61205	40100 - 51062	29865 - 40099	19405 - 29864	9291 - 19404
2(EV)001	2(EZ)001	2(EZ)001	2(EU)001	3(ELU)001	2(EZ)003	2(FU)001	2(EU)001
Note to repeat 1: Sall box (V) is 1054 bp long			Note to repeat 4: Sall box U is 1046 bp long				

F1J10

1	2	3	4	5	6	7	8	9
6579 - 18122	18122 - 28583	28584 - 38727	38728 - 49771	49772 - 39303	59304 - 69670	69671 - 80276	80277 - 90410	90411 - 101368
4(EEET)001	2(EV)001	2(EV)001	2(EV)001	2(EU)001	2(EZ)001	2(EZ)001	2(EU)001	3(ELU)001

F2I8

1	2	3	4	5	6	7	8	9
84706 - 95227	74230 - 84705	63349 - 74229	53500 - 63648	44049 - 53499	33608 - 44048	23068 - 33607	13619 23067	3183 - 13618
2(FV)001	2(EV)001	2(EZ)003	2(FQ)003	2(EC)003	2(EU)111	2(EY)003	2(EC)003	2(EU)111

F2J3

1	2	3	4	5	6	7	8	9
12334 - 22270	22271 - 32754	32755 - 42020	42021 - 52162	52163 - 61314	61315 - 70586	70587 - 80790	80791 - 91243	91214 - 100490
2(E&)003	2(EV)001	1(M)003	2(FQ)013	1(I)003	1(I)001	2(EP)001	2(EU)001	1(M)003

F2M18

1	2	3	4	5	6	7	8
75695 - 86275	65553 - 75694	55408 - 65552	44932 - 55407	34581 - 44931	25203 - 34580	14900 - 25202	4462 - 14899
2(EZ)003	2(FQ)003	2(FQ)013	2(EV)101	2(DT)001	1(N)003	2(ER)111	2(EU)001
	Note to repeat 2: Sall box (F) is 308 bp		Note to repeat 4: Sall box(V) is 1054 bp long				

F2L18

1	2	3	4	5	6	7
60653 - 70811	51381 - 60652	41435 - 51380	31500 - 41434	22219 - 31499	11761 - 22218	2851 - 11760

2(FQ)013	1(I)001	2(E&)003	2(E&)003	1(I)001	2(EU)001	1(M)013D

F2L21

1	2	3	4	5	6
6432 - 16519	16520 - 26796	26797 - 37278	37279 - 47737	47338 - 57990	57991 - 68430
2(FQ)113	2(FQ)111	2(EV)??1	2(DU)001	2(DQ)001	2(EU)001

F2M10

1	2	3	4	5	6
55799 - 66888	44711 - 55798	33642 - 44710	22592 - 33641	11543 - 22591	1828 - 11542
3(EEV)111	3(EEV)001	3(EEU)111	3(EEU)001	3(EEU)001	3(EEU)00?
Note to repeat 1: [illegible]	Note to repeat 2: Sall box (V) is 1051 bp long				

F2M12

1	2	3	4	5	6	7	8	9
16157 - 26646	26647 - 35926	35927 - 46040	46041 - 55303	55304 - 65718	65719 - 74631	74632 - 84715	84716 - 93970	93971 - 104007
2(EV)001	1(M)003	2(FQ)003	1(I)001	2(EU)001	1(M)003D	2(FQ)013	1(I)001	2(E&)001
				Note to repeat 5: Sall box (U) is 1000 bp long			Note to repeat 8: Sall box (I) is 450 bp long	

F2M13

1	2	3	4	5	6
55861 - 66397	45382 - 55860	37703 - 45381	23930 - 34702	13491 - 23929	2833 - 13490
2(FV)111	2(EV)111	2(EZ)001	2(?U)001	2(EU)001	2(EY)001
	Note to repeat 2: Sall box (V) is 1053 bp long				

F2O12

1	2	3	4	5	6	7
58302 - 67730	48447 - 58501	37529 - 48446	27047 - 37528	17650 - 27046	7177 - 17649	71776 - ????
1(H)001	2(E&)001	2(EΨ)001	2(EV)011	1(M)001	2(EU)011	2(EZ)???
			Note to repeat 4: Sall box (V) is 1054 bp long			Note to repeat 7: the repeat is incomplete

F2N4

1	2	3	4	5	6
49965 - 60649	39255 - 49964	28581 - 39254	17886 - 28580	7211 - 17885	17884 - ???
2(EZ)001	2(EZ)001	2(EZ)001	2(EZ)001	2(EZ)001	2(EZ)???
					Note to repeat 6: the repeat is incomplete

F2P1

1	2	3	4	5
10075 - 20585	20587 - 31063	31064 - 41529	41530 - 52090	52091 - 62570
2(FV)001	2(EV)001	2(EU)001	2(EZ)003	2(FU)001
Note to repeat 1: [illegible]	Note to repeat 2: Sall (V) is 1052 bp long			

F1N7

1	2	3	4
12524 - 23626	23627 - 34717	34718 - 45803	45804 - 56875
3(EEV)111	3(EEV)001	3(EEV)001	3(EEU)111

F1C17

1	2	3	4
16069 - 26342	26343 - 36824	36825 - 47004	47005 - 57674

	2(FQ)001	2(EV)001	2(EU)001C	2(EZ)001		
F2L11						
	1	2	3	4	5	
	16705 - 25863	25864 - 35327	35328 - 45365	45366 - 54619	54620 - 64520	
	1(I)003	1(I)003A	2(FO)013	1(I)001	2(E&)001	
F2O18						
	1	2	3	4	5	
	16358 - 26940	26941 - 37082	37083 - 472256	47227 - 57692	57693 - 67177	
	2(EZ)003	2(FQ)003	2(FQ)013	2(EU)001	2(FU)001C	
F2J21						
	1	2	3	4		
	17457 - 26568	26569 - 35677	35678 - 44945	44946 - 54201		
	1(E)001	1(E)011	1(M)013	1(H)111		
F2J9						
	1	2	3	4	5	6
	12360 - 22759	22760 - 33706	33707 - 44412	44413 - 55332	55333 - 66020	66021 - 75977
	2(EZ)001A	2(Eλ)001	2(EZ)001	2(EΨ)001	2(EZ)001	2(EZ)001
F16K18						
	1	2	3	4	5	
	43376 - 53866	33211 - 43375	22706 - 33210	13323 - 22705	2882 - 13322	
	2(EV)001	2(EV)001	2(EV)001	1(M)011	2(EU)001	
F17A7						
	1	2	3	4		
	42038 - 53132	30938 - 42037	19839 - 30937	8755 - 19838		
	3(EEV)001	3(EEV)111	3(EEV)111	3(EEU)111		
F19A6						
	1	2	3	4	5	6
	6200 - 15477	15478 - 24460	24461 - 33888	33889 - 43614	43615 - 52883	52884 - 62084
	1(I)111	1(M)013	1(N)111	1(U)001	1(I)111	1(L)011
F16L16						
	1	2	3			
	27304 - 37643	17151 - 27303	6987 - 17150			
	2(EZ)001	2(EU)001	2(EU)001			
F16L21						
	1	2	3			
	35506 - 46080	46081 - 56481	56482 - 66877			
	2(IV)111	2(EV)111	2(EU)111			
F16P19						
	1	2	3	4		
	6565 - 16969	16970 - 27406	27407 - 37831	37832 - 48222		

2(EV)001	2(EV)001	2(EV)001	2(EU)001	

F18E22

1	2	3	4	5	6	7
16466 - 26908	26909 - 36309	36310 - 46466	46467 - 56947	56948 - 67405	67406 - 77383	77383 - 87934
2(EV)001	1(O)003	2(FQ)003	2(EV)111	2(EU)001	2(ER)001C	2(EZ)003

F18E11

1	2	3	4	5	6
52693 - 62668	42657 - 52692	32616 - 42656	23348 - 32615	13303 - 23347	2799 - 13302
1(Z)003	2(EO)003	2(EO)013	1(I)001	2(E&)001	2(EV)001

F18C10

1	2	3	4
39352 - 49572	29277 - 39351	19023 - 29276	8627 - 19022
2(EV)001	2(EU)003	2(FQ)001	2(EU)001

F2O4

1	2	3	4
59926 - 70615	50533 - 59952	40069 - 50532	30704 - 40068
2(EZ)001	1(M)011	2(ET)001	1(M)001
