# Supplementary material for: Sequencing of the Arabidopsis NOR2 reveals its distinct organization and tissue-specific rRNA ribosomal variants
Source: Nat Commun. 2021 Jan 15;12:387. doi: 10.1038/s41467-020-20728-6 (PMC7810690; doi:10.1038/s41467-020-20728-6)
Supplement: Supplementary file 2 — Peer Review [file 41467_2020_20728_MOESM2_ESM.pdf]

Reviewer #1 (Remarks to the Author):

The authors of the submitted manuscript "Sequencing and analysis of Arabidopsis thaliana NOR2 reveal its distinct organization and tissue-specific expression of rRNA ribosomal variants" took advantage of the recent progress in genomics and DNA sequencing techniques to shed new light on our understanding of fine molecular organization of ribosomal DNA loci, its regulation and functioning. The ribosomal DNA (rDNA) plays pivotal part across all advanced forms of life by encoding four ribosomal RNAs which are the major structural and functional components of ribosome, the essential protein synthesis machinery. To ensure sufficient supply of ribosomal RNAs, the rRNA genes are present in thousands copies per genome in most of plant species. The high copy number of rRNA genes per genome, was one of the reasons that they were among first sequenced and broadly studied eukaryotic genes. Simultaneously, this poses additional problems for understanding regulation of the rRNA genes expression, especially considering certain level of the genes sequence heterogeneity and their localization at multiple chromosomal loci.
While the organization of individual 45S rDNA units coding for 18- 5.8- 25S rRNAs, has been well documented for many eukaryotes including multiple plant species, the knowledge on organization of the 45S rDNA loci is rather limited even in our age of massive genome sequencing because of intrinsic limitations of the current genome sequencing techniques unable to resolve repetitive DNA regions. Submitted study addressed the problem by analysing 45S rDNA locus composition in model plant Arabidopsis, which contains relatively low number of rDNA repeats for plants, about 800 copies distributed between two loci, through checking a collection of selected BAC clones using Nanopore long-reads and Illumina sequencing. The obtained raw data was subsequently subjected to detailed sorting by sophisticated marker system combined with various bioinformatics tools, some developed in-house. This approach allows to demonstrate a significant heterogeneity of the 45S rDNA repeats within the locus and to conclude that the rDNA variants are distributed not randomly, but are organized in clusters. Those not very new ideas have been expressed previously, but have never been experimentally confirmed with such a clarity and certainty. Another important highlight of the study is a discovery that different variants of rRNA genes found in the 45S rDNA loci clusters, are diferentially integrated into the functional ribosomes in different plant tissues.

The study's findings for the model Arabidopsis plant will definitely stimulate further research on the rDNA chromosomal arrangement, regulation and functionality in other plant species, including major crops due to rRNA fundamental role in multiple processes related to plant development and productivity. The methods and approaches used in the study are described with care and details, making it easy to adopt for other species. Moreover, the uncovered principles could be exploited also in mammalian systems including human. For example, a number of health disorders in humans have been related to rDNA aberrations; establishing associations between certain rDNA rearrangements and/or specific ribosomal rRNA variants with an illness, could potentially lead to developing drugs for curing the disease.

While the manuscript is generally well written and illustrated, I would suggest a couple of updates to further improve the presentation and understanding of the presented data:
- In Abstract, it reads "We used a combination of long- and short-read sequencing technologies to assemble contigs of the Arabidopsis NOR2 rDNA domain providing a first map" (lines 20-21). I did not find the claimed map in the manuscript, either it should be included or the statement removed.
- Lines 24-25: "… and provide the higher order organization of NOR2." I suggest to modify the statement to : "… and provide the insights into higher order organization of NOR2."
- Line 76: "In total we sequenced 59 BACs with an average BAC assembly size…". I suppose authors refer to long-read Nanopore sequencing, should be clarified.
- Lines 172-174: "The quality and length of the contigs generated provided a first map of NOR2 that could be analyzed for its higher-order organization albeit of being incomplete". To my view, generation of relatively long contigs (230 and 200 kb long, lines 168-169) is the backbone of current study. The contigs arrangement should be schematically presented as a main illustration, not just saying it "provided a first map of NOR2", which it basically does not. The two longest assembled contigs cover

just about 10% of the locus (~40 rDNA repeats out of about 400 repeats in the NOR2).
- It would be helpful to put fragment size markers for Figure S1b.

Reviewer #2 (Remarks to the Author):

The authors built the first NOR sequence draft based on the MinION sequencing of 59 BAC clones that represent a valuable resource to researchers who are interested. They also difined the sequence variations in rRNA genes and investigated the tissue-specific gene expression manner. Undoubtly, the data provides a higher order NOR organization, arriving 80 kb in length and 10 copies of rDNA for the first time.
We all know that the model genome of Arabidopsis is comprehensively studied since 2000. Here, the selected 59 BACs don't cover the full NOR2 region, so the discontigous BACs is destined to getting incomplete and mostly unassembled NORs, whereas the project aim is "to generate a complete assembly of the NOR2" that is stated in their results. The whole genome sequencing is advantageous to harvest the full NOR with the existence of all DNA if the sequence read is long enough to differentiate the repetative sequences from each other. Whether the BAC-based sequencing or the whole genome way is better for NORs needs to be discussed. The BAC-based way generates 53 contigs with an average length of 80 kb for NOR2 in this paper, but leaving the low amount of overlaps between these BACs. A report of seven Arabidopsis thaliana genome assemblies are shown to have parts of the highly complex regions of centromeres, telomeres and rDNA clusters (Jiao, W., Schneeberger, K. Nat Commun 11,2020). The chromosome-level assembly of Arabidopsis thaliana Ler reveals NOR fragments too (Luis Zapata et al. 2016. PNAS). On the other hand, the inherent repetative regions might be never achieve this goal by simply whole-genome assembly. The paper (Michael, et al. 2018, NC) is not able to identify the NOR on the short arms of chromosome 2 and 4 using one Nanopore sequencing cell.
I believe the newly assembled Arabidopsis genome generated by PacBio platform also includes the partial NOR2 regions, but is harder to piece them together. I am very curious about the NOR assembly comparison between whole genome assembly and the BAC version. It is generally accepted that the Arabidopsis reference genome contains collapsed regions where tandem repeats such as rDNA could not be resolved by the BAC-based Sanger method. It is promising that the program of OverBACer could help determine the overlapping BACs by using a string of marker identities. One addition that I think would boost the impact of the study would be a try to fish out all rDNA reads from whole genome sequencing done by Pacbio or Nanopore, assemble them and merge the contigs into large contigs by OverBACer.
Overall, they generate a first verion of NOR, identify sequence variation and gene expression features that would benefit scientists for their further studies. Still, the missing sequences and the unkown mechanisms for high copies of rDNA and sequence variation didn't significantly imporve our understanding in NOR function.

There are some small typos and minor suggestions that the authors might consider, which I noted below:
1) If more about the development of NOR sequencing or the limitation in the current Arabidopsis genome assembly is given in the introduction, it will be easily realized how chanllenge it is to retrieve the NOR regions.
2) The Results is mingled too much details of Methods. For example, line 84-95 is better to be moved to Methods because it just shows how Illumina reads are mapped and how the assembly is validated by certain enzymes.
3) It is good to explain everything about the experimental steps that allows to be replicated. Still, the routine details like about the Illumina and Nanopore sequencing except the unique barcode system can be skipped.
4) The Discussion is more like a summary of Result. Here, the further discusstion about how NORs

function in plant genomes or in other kingdoms are expected.
5) Line 130-133 is redundant of Line 181-183.
6) In Figure 4: “SalI” is a typo.
7) In Figure 5d: “DNA” in the lengend is not consistent with “WG” in the figure.
8) Fig S1a: Should the label of “var2” be removed because these clones belong to NOR2?
9) In Fig S1c: ”lenght” is a typo.
10) Suppl. Table 3: “Unequal distribution of SNP/InDels between NOR2 and NOR4” should be corrected into “Unequal distribution of SNP/InDels between NOR2 and the whole genome”.
11) Line 233:Figure 4d need be corrected into Figure 3d.
12) Line 234:Figure 4c need be corrected into Figure 3c.
13) Line 300:Figure 6d need be corrected into Figure 5d.
14) Although all sequencing data is deposited under SRA BioProject number PRJNA632576 and PRJNA555773, they are not public yet.

Reviewer #1 (Remarks to the Author):

The authors of the submitted manuscript “Sequencing and analysis of Arabidopsis thaliana NOR2 reveal its distinct organization and tissue-specific expression of rRNA ribosomal variants” took advantage of the recent progress in genomics and DNA sequencing techniques to shed new light on our understanding of fine molecular organization of ribosomal DNA loci, its regulation and functioning. The ribosomal DNA (rDNA) plays pivotal part across all advanced forms of life by encoding four ribosomal RNAs which are the major structural and functional components of ribosome, the essential protein synthesis machinery. To ensure sufficient supply of ribosomal RNAs, the rRNA genes are present in thousands copies per genome in most of plant species. The high copy number of rRNA genes per genome, was one of the reasons that they were among first sequenced and broadly studied eukaryotic genes. Simultaneously, this poses additional problems for understanding regulation of the rRNA genes expression, especially considering certain level of the genes sequence heterogeneity and their localization at multiple chromosomal loci.
While the organization of individual 45S rDNA units coding for 18- 5.8- 25S rRNAs, has been well documented for many eukaryotes including multiple plant species, the knowledge on organization of the 45S rDNA loci is rather limited even in our age of massive genome sequencing because of intrinsic limitations of the current genome sequencing techniques unable to resolve repetitive DNA regions.
Submitted study addressed the problem by analysing 45S rDNA locus composition in model plant Arabidopsis, which contains relatively low number of rDNA repeats for plants, about 800 copies distributed between two loci, through checking a collection of selected BAC clones using Nanopore long-reads and Illumina sequencing. The obtained raw data was subsequently subjected to detailed sorting by sophisticated marker system combined with various bioinformatics tools, some developed in-house. This approach allows to demonstrate a significant heterogeneity of the 45S rDNA repeats within the locus and to conclude that the rDNA variants are distributed not randomly, but are organized in clusters. Those not very new ideas have been expressed previously, but have never been experimentally confirmed with such a clarity and certainty. Another important highlight of the study is a discovery that different variants of rRNA genes found in the 45S rDNA loci clusters, are diferentially integrated into the functional ribosomes in different plant tissues.

The study’s findings for the model Arabidopsis plant will definitely stimulate further research on the rDNA chromosomal arrangement, regulation and functionality in other plant species, including major crops due to rRNA fundamental role in multiple processes related to plant development and productivity. The methods and approaches used in the study are described with care and details, making it easy to adopt for other species. Moreover, the uncovered principles could be exploited also in mammalian systems including human. For example, a number of health disorders in humans have been related to rDNA aberrations; establishing associations between certain rDNA rearrangements and/or specific ribosomal rRNA variants with an illness, could potentially lead to developing drugs for curing the disease.

We are more than delighted to read the comments of “Reviewer #1”. We are grateful for her/his appreciation of the study.

While the manuscript is generally well written and illustrated, I would suggest a couple of updates to further improve the presentation and understanding of the presented data:
- In Abstract, it reads “We used a combination of long- and short-read sequencing technologies to assemble contigs of the Arabidopsis NOR2 rDNA domain providing a first map” (lines 20-21). I did not find the claimed map in the manuscript, either it should be included or the statement removed.

We have removed this statement as suggested by the reviewer

- Lines 24-25: “… and provide the higher order organization of NOR2.” I suggest to modify the statement to : “… and provide the insights into higher order organization of NOR2.”

We have modified the statement as suggested by the reviewer

- Line 76: "In total we sequenced 59 BACs with an average BAC assembly size…". I suppose authors refer to long-read Nanopore sequencing, should be clarified.

We have clarified this sentence by adding "In total we sequenced 59 BACs with an average BAC assembly size of about 77 kb (Supplementary Table 1) with Oxford Nanopore Technologies "

- Lines 172-174: "The quality and length of the contigs generated provided a first map of NOR2 that could be analyzed for its higher-order organization albeit of being incomplete". To my view, generation of relatively long contigs (230 and 200 kb long, lines 168-169) is the backbone of current study. The contigs arrangement should be schematically presented as a main illustration, not just saying it "provided a first map of NOR2", which it basically does not. The two longest assembled contigs cover just about 10% of the locus (~40 rDNA repeats out of about 400 repeats in the NOR2).

Following the reviewer recommendation we have added a schematic representation of the BAC overlaps in Figure 1.

- It would be helpful to put fragment size markers for Figure S1b.

We have added a fragment size marker in Figure S1b

Reviewer #2 (Remarks to the Author):

The authors built the first NOR sequence draft based on the MinION sequencing of 59 BAC clones that represent a valuable resource to researchers who are interested. They also difined the sequence variations in rRNA genes and investigated the tissue-specific gene expression manner. Undoubtly, the data provides a higher order NOR organization, arriving 80 kb in length and 10 copies of rDNA for the first time.
We all know that the model genome of Arabidopsis is comprehensively studied since 2000. Here, the selected 59 BACs don't cover the full NOR2 region, so the discontigous BACs is destined to getting incomplete and mostly unassembled NORs, whereas the project aim is "to generate a complete assembly of the NOR2" that is stated in their results. The whole genome sequencing is advantageous to harvest the full NOR with the existence of all DNA if the sequence read is long enough to differentiate the repetative sequences from each other. Whether the BAC-based sequencing or the whole genome way is better for NORs needs to be discussed. The BAC-based way generates 53 contigs with an average length of 80 kb for NOR2 in this paper, but leaving the low amount of overlaps between these BACs. A report of seven Arabidopsis thaliana genome assemblies are shown to have parts of the highly complex regions of centromeres, telomeres and rDNA clusters (Jiao, W., Schneeberger, K. Nat Commun 11,2020). The chromosome-level assembly of Arabidopsis thaliana Ler reveals NOR fragments too (Luis Zapata et al. 2016. PNAS). On the other hand, the inherent repetative regions might be never achieve this goal by simply whole-genome assembly. The paper (Michael, et al. 2018, NC) is not able to identify the NOR on the short arms of chromosome 2 and 4 using one Nanopore sequencing cell.
I believe the newly assembled Arabidopsis genome generated by PacBio platform also includes the partial NOR2 regions, but is harder to piece them together. I am very curious about the NOR assembly comparison between whole genome assembly and the BAC version. It is generally accepted that the Arabidopsis reference genome contains collapsed regions where tandem repeats such as rDNA could not be resolved by the BAC-based Sanger method. It is promising that the program of OverBACer could help determine the overlapping BACs by using a string of marker identities. One addition that I think would boost the impact of the study would be a try to fish out all rDNA reads from whole genome sequencing done by Pacbio or Nanopore, assemble them and merge the contigs into large contigs by OverBACer.
Overall, they generate a first verion of NOR, identify sequence variation and gene expression features that would benefit scientists for their further studies. Still, the missing sequences and the unkown mechanisms for high copies of rDNA and sequence variation didn't significantly imporve our understanding in NOR function.

We are very thankful to Reviewer #2 for the critical reading of our manuscript, for raising very important points and for stimulating interesting additional approaches. Inspired by his suggestion to use NanoPore reads from whole genome sequencing runs to build the entire NOR2 (with the aid of OverBACer), we used the publicly available datasets of (Michael, et al. 2018, NC) and merged 4 NanoPore runs for a total of ~ 2 M reads (~120 x coverage of the genome). Unfortunately the PacBio reads were not suitable to assemble the NORs since they are shorter and of lower quality than the NanoPore reads. From this large dataset we performed 4 different assemblies. The first two assemblies were carried out without filtering for reads that map to the rDNA. We reasoned that by having the entire *Arabidopsis* genome, the assembler could use the unique sequences of chromosome 2 as anchor points to

assemble NOR2. The first assembly was performed with all reads and the second with reads longer than 30 kb. Only 7 contigs per assembly contained rDNA units and of these only 2 contigs were longer than 40 kb (~ 4 repeats).
We next performed 2 assemblies with NanoPore reads filtered for the presence of rDNA. As for the previous experiments, one assembly was performed with all reads and one only with reads longer than 30 kb. For both cases only 6 contigs assembled, with only 4 longer than 40 kb. Furthermore, the assembled rDNA contigs contained a mixture of VAR1, VAR2 and VAR3 (representing rDNA units from both, chromosome 2 and 4) suggesting that these contigs represent assembly artefacts.

	Mean Quality	n of Reads	Mean read length	n of bases	n of rDNA contigs assembled	rDNA contigs longer than 40kb	n of rDNA units
All reads	8.5	2 M	8 K	16 Gb	7	2	7
Reads ≥30kb ≥q8	9.1	100 K	30 K	3 Gb	7	2	7
rDNA reads ≥30kb	7.4	4 K	40 K	158 Mb	6	4	15
rDNA reads ≥30kb ≥q8	9.6	1 K	40 K	43 Mb	6	4	14

The use of BACs to partially reconstruct NOR2 has the advantage that for each BAC sequenced we can determine the physical length and estimate the number of units per BAC by Pulse Field Electrophoreses. In addition, we can assemble each BAC by using several ultra-long reads that cover the entire length of the BAC (~ 100 kb). This is unfortunately not feasible when running whole genome sequencing experiments since the NORs are estimated to be 4 Mb in length and we would need multiple ultra-long reads (~ 4 Mb) to successfully assemble them. Currently ultra-long reads are not available in sufficient length and number to unambiguously reconstruct the rDNA regions on chromosome 2 and 4 directly from plant DNA. The fast pace at which the sequencing technology is being developed will likely provide tools to directly sequence large repetitive regions from plants in the near future.

Furthermore, following the suggestions of Reviewer #2 we have changed the sentence of our project aim "to generate a complete assembly of NOR2" to "to generate a draft assembly of NOR2" (Line 75 of revised manuscript).

There are some small typos and minor suggestions that the authors might consider, which I noted below:
1) If more about the development of NOR sequencing or the limitation in the current Arabidopsis genome assembly is given in the introduction, it will be easily realized how chanllenge it is to retrieve the NOR regions.

We agree with Reviewer #2 and we added a few sentences to exemplify the challenging aspects of assembling the NORs: "The current Arabidopsis genome assembly only contains single rDNA units at the top of chromosomes 2 and 4. Furthermore, none of the recent Arabidopsis thaliana genome assembly approaches 10,11, performed with devices and protocols from Oxford Nanopore Technologies or PacBio, provided contigs with multiple 45S rDNA units in tandem. The difficulty in assembling the NORs, from whole genome sequencing projects, is intrinsic to the low complexity of the nearly identical rDNA units. Currently ultra-long reads are not available in sufficient length and number to unambiguously reconstruct the rDNA regions on chromosome 2 and 4 directly from plant DNA." (Lines 51-58 of revised manuscript).

2) The Results is mingled too much details of Methods. For example, line 84-95 is better to be moved to Methods because it just shows how Illumina reads are mapped and how the assembly is validated by certain enzymes.
3) It is good to explain everything about the experimental steps that allows to be replicated. Still, the routine details like about the Illumina and Nanopore sequencing except the unique barcode system can be skipped.

We agree with Reviewer #2 and moved the following sections of the results to the Material and Methods. The paragraph is now located at Lines: 762-773 of the revised manuscript.

4) The Discussion is more like a summary of Result. Here, the further discusstion about how NORs function in plant genomes or in other kingdoms are expected.

Following the suggestions of Reviewer #2 we have added multiple sections in the discussion comparing the function of the *Arabidopsis* NOR2 with that of other organisms. Lines 331-340, Lines 365-368 and Lines 381-385 of the revised manuscript.

5) Line 130-133 is redundant of Line 181-183.

We have removed the redundant line 181-183 and substitute it with "four marker barcode system" (Line 182 of the revised manuscript)

6) In Figure 4: “SalI” is a typo.

We have corrected the *SalI* typo

7) In Figure 5d: “DNA” in the lengend is not consistent with “WG” in the figure.

We have adjusted the typo in Figure 5d

8) Fig S1a: Should the label of “var2” be removed because these clones belong to NOR2?

In Figure S1a the gel contains genotyped BACs also with VAR2. This is just to show that it is possible to discriminate each BAC based on the VAR contained in the rDNA units. For this reason we believe it is important to keep the Var2 label.

9) In Fig S1c: ”lenght” is a typo.

We have corrected the typo

10) Suppl. Table 3: “Unequal distribution of SNP/InDels between NOR2 and NOR4” should be corrected into “Unequal distribution of SNP/InDels between NOR2 and the whole genome”.

We have changed the sentence based on the reviewer suggestions

11) Line 233:Figure 4d need be corrected into Figure 3d.
12) Line 234:Figure 4c need be corrected into Figure 3c.
13) Line 300:Figure 6d need be corrected into Figure 5d.

We have corrected all typos suggested by the Reviewer

14) Although all sequencing data is deposited under SRA BioProject number PRJNA632576 and PRJNA555773, they are not public yet.

We have made the sequencing data and the assembled contigs publicly available

All changes made to the manuscript are highlighted in the text file.

Reviewer #2 (Remarks to the Author):

It is nice that the authors have validated the BAC-dependant approach is better than the way of whole genome sequencing by using four runs of assembly strategies. They further highlighted the challenges and significance of NOR organization. I think that my concerns have been largely addressed. Although the paragraph of result “Evalution of the Assembly” was moved to Methods, the words need be deleted ((see Materials & Methods), (Supplementary Table 2), (Supplementary Fig. 1d)…).
Wenqin Wang

REVIEWER COMMENTS / **ANSWERS TO THE REVIEWER**

It is nice that the authors have validated the BAC-dependant approach is better than the way of whole genome sequencing by using four runs of assembly strategies. They further highlighted the challenges and significance of NOR organization. I think that my concerns have been largely addressed. Although the paragraph of result "Evalution of the Assembly" was moved to Methods, the words need be deleted ((see Materials & Methods), (Supplementary Table 2), (Supplementary Fig. 1d)…).
Wenqin Wang

We are more than delighted to read the comments of Reviewer #2. We are grateful for her appreciation of the study. As suggested by the reviewer we have removed the advised words.
